# E3 ligase CHIP restoration facilitates the effect of α_1_-adrenoceptor blockage on alleviating lipopolysaccharide-caused cardiac fibrosis via downregulating TGF-BR1 expression and Smad2/3 activation

**DOI:** 10.1186/s43556-025-00357-5

**Published:** 2025-11-10

**Authors:** Wan Lin, Hang Li, Hin Fong, Xingyu Su, Junhao Wen, Xianyun Shao, Ziqing Yan, Yiyang Wang

**Affiliations:** Department of Pathophysiology, School of Medicine, Jinan University, Guangzhou, Guangdong 510632 China

**Keywords:** Lipopolysaccharide, Cardiac fibrosis, Fibroblast, Norepinephrine, α_1_-AR, CHIP

## Abstract

Myocardial fibrosis is a serious complication in sepsis and leads to cardiac dysfunction. The carboxy terminus of Hsc70-interacting protein (CHIP), a U-box E3 ligase, defends against sepsis-caused cardiac injury. Here, we explored a novel therapeutic effect of α_1_-adrenoceptor (α_1_-AR) blockage on lipopolysaccharide (LPS)-induced myocardial fibrosis and clarified that its molecular mechanism was related to the restoration of CHIP expression. The results showed that LPS increased the release of norepinephrine (NE) in the myocardium and promoted myocardial fibrosis. NE promoted the cardiac fibroblasts (CFs) differentiation characterized by increased α-SMA and collagen I/III. Blockage of α_1_-AR by prazosin apparently alleviated LPS-induced cardiac fibrosis and NE-caused CFs differentiation. Prazosin decreased phosphorylation of protein kinase C (PKC), p38 and Smad2/3, and reduced nuclear c-Jun level, as well as increased CHIP expression in the NE-stimulated CFs and the myocardium in LPS-treated mice. In vitro and in vivo data suggested that the overexpression of CHIP restrained α-SMA and collagen I/III production, and downregulated TGF-β receptor 1 (TGF-BR1) expression and Smad2/3 phosphorylation induced by NE or LPS respectively. Conversely, the knockdown of CHIP weakened the effect of prazosin. Furthermore, we innovatively revealed that α_1A_-AR is the dominant α_1_-AR subtype in CFs, and its specific antagonist, silodosin, eliminated NE-mediated CFs differentiation and LPS-induced myocardial fibrosis, which was consistent with the action of prazosin. These findings demonstrate the protective effect of α_1_-AR blockage against LPS-mediated myocardial fibrosis, which is achieved by directly inhibiting the PKC-p38-Smad2/3 signaling pathway and promoting TGF-BR1 downregulation through restoring CHIP expression.

**Supplementary Information:**

The online version contains supplementary material available at 10.1186/s43556-025-00357-5.

## Introduction

Sepsis, as a severe inflammatory disease, is a dysregulated host response to infection, which leads to life-threatening multiple organ dysfunction [1]. As a common complication of sepsis, cardiac dysfunction is associated with the significantly increased in mortality [2, 3]. The investigations involving animal models and human patients have indicated that lipopolysaccharide (LPS) plays a pivotal role in the pathogenesis of cardiac dysfunction during sepsis [4, 5]. LPS induces myocardial fibrosis, which attenuates the systolic and diastolic functions of the heart, thereby promoting the occurrence of congestive heart failure [6]. Therefore, it is of great importance to explore effective anti-myocardial fibrosis theoretical strategies for improving sepsis-induced cardiac dysfunction.

Cardiac fibroblasts (CFs), which account for 15 to 20% of the cells in the heart tissue [7], are activated and subsequently differentiate into myofibroblasts after initial injury that synthesize more collagen [8–11]. The studies reported that myocardial fibrosis exerted an important effect on LPS-induced cardiac dysfunction, which is a typical characteristic of pathological remodeling and heart failure [12, 13]. It has been shown that the transforming growth factor-beta (TGF-β) pathway activation mediated cardiac fibrosis by inducing the differentiation of fibroblasts to myofibroblasts [14]. Specifically, TGF-β enhances pro-fibrotic signaling via binding to TGF-β receptor 2 (TGF-BR2), which recruits and activates TGF-β receptor 1 (TGF-BR1) and subsequently leads to activation of the canonical Smad2/3 signaling pathway [15]. Therefore, suppression of the TGF-β/Smad2/3 pathway in cardiac fibroblasts may improve sepsis-associated cardiac fibrosis and also hinder cardiac dysfunction.

Research has reported that systemic levels of norepinephrine (NE) are increased during sepsis [16–18]. Although, during sepsis, the involvement of LPS-induced inflammatory cytokine production in the progression of cardiac fibrosis has been well investigated [19], the effect and intrinsic mechanisms of the NE-mediated signaling pathway on myocardial fibrosis are not yet fully understood. The experimental report indicated that NE-induced remodeling of the left ventricle of the rat heart resulted in interstitial fibrosis [20]. Although there are α-adrenoceptor (α-AR) and β-adrenoceptor (β-AR) in the myocardial tissue, due to the more potent binding with α-AR [21, 22], NE is likely to increase the collagen expression in CFs through activating α-AR [23]. Our own work has demonstrated that α_2_-AR activation accelerated LPS-induced cardiac fibrosis and CFs differentiation [24]. However, the role of α_1_-AR in sepsis-associated fibrosis has not been well described. Study in mice found that α_1_-AR blockade with prazosin prevented cytokine storm and markedly improved survival in LPS-challenged mice [25]. The α_1_-AR participated in myocardial hypertrophy through protein kinase C (PKC)-mediated cell anabolism and growth [26]. Recent research suggested that NE aggravated TGF-β-induced renal epithelial-mesenchymal transition via the α_1_-AR/p38/Smad3 axis and the blockade of α_1_-AR alleviated renal fibrosis in mice [27]. Therefore, we hypothesized that increased NE in myocardial tissue may promote cardiac fibrosis during sepsis by activating α_1_-AR in the CFs, and blockage of α_1_-AR may alleviate myocardial fibrosis caused by NE, resulting in improvement of the cardiac function.

The carboxyl terminus of HSP70-interacting protein (CHIP), as a U-box E3 ubiquitin ligase, was reported to control the sensitivity of TGF-β signaling by promoting Smad3 degradation [28, 29]. It suggests that CHIP has a potential anti-fibrotic function. Our previous study indicated that LPS down-regulated CHIP expression in cardiomyocytes through activating transcription factor c-Jun [30]. We speculate that NE-mediated α_1_-AR activation may inhibit CHIP expression in CFs by activating the PKC/p38/c-Jun axis, which promotes myocardial fibrosis during sepsis.

Therefore, we propose the hypothesis that blockage of α_1_-AR may directly inhibit the activation of NE-mediated pro-fibrotic pathway and restore CHIP expression through eliminating the inhibitory effect of NE on CHIP, thereby alleviating sepsis-caused myocardial fibrosis. This study revealed the anti-myocardial fibrosis effect of prazosin in LPS-stimulated mice. We provided convincing evidence supporting the hypothesis that blockage of α_1_-AR not only inhibited the PKC-p38-Smad2/3 pathway, but also up-regulated CHIP expression in CFs stimulated by NE. Up-regulation of CHIP downregulated TGF-BR1 expression through ubiquitin-modification and reduced the activation of Smad2/3, resulting in inhibiting the NE-induced differentiation of CFs and LPS-caused cardiac fibrosis. Moreover, we found that the α_1A_-AR subtype is highly expressed in CFs. Blockage of α_1A_-AR by silodosin produced a therapeutic effect similar to that of prazosin in preventing LPS-induced myocardial fibrosis. Our research presents novel insights that antagonists targeting α_1_-AR and its subtype alleviate LPS-associated myocardial fibrosis, which may be an attractive therapeutic strategy for cardiac dysfunction in patients with sepsis.

## Results

### Blockage of α_1_-AR improved cardiac fibrosis and dysfunction in the mice after LPS stimulation

**Fig. 1:**
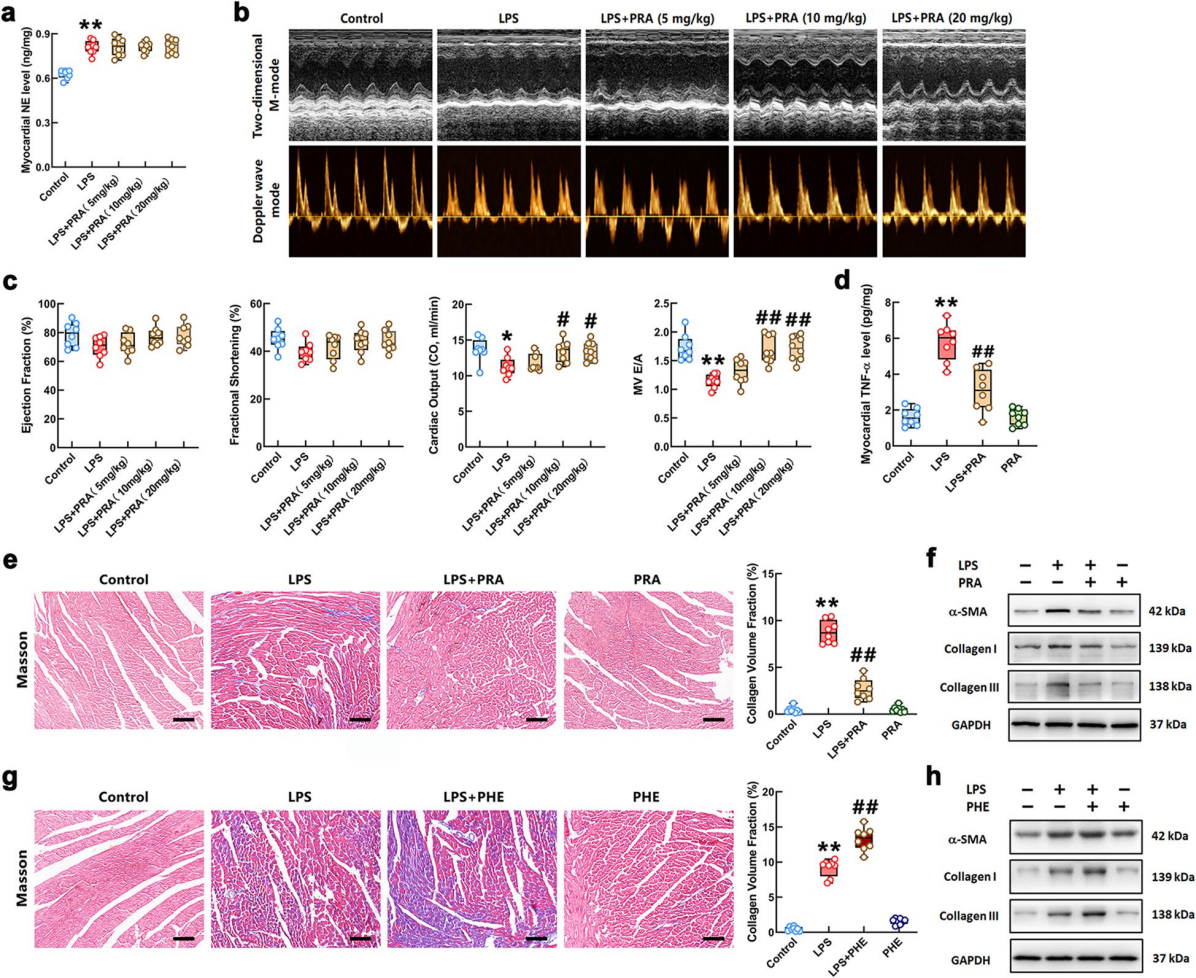
Blockage of α_1_-AR improved cardiac dysfunction and fibrosis in the mice with LPS stimulation. **a** NE content in the myocardium of mice was measured by ELISA. **b** Representative echocardiograms of M-mode (upper) and Doppler wave mode (bottom) of mice treated with LPS and different doses of PRA. **c** Quantitative group data for echocardiographic analyses: ejection fraction, fractional shortening, cardiac output and E/A ratio. **d** The TNF-α level of the cardiac tissues in the mice was measured by ELISA. **e** and **g** Representative Masson's trichrome staining images of myocardial sections (Scale bar = 100 μm.) and quantification of the fractional fibrotic area in different groups. Red staining represents myofiber and blue staining represents collagen fiber. **f** and **h** The protein levels of α-SMA and collagen I/III in the myocardium of mice were measured by Western Blot in each group. *n* = 6 or 8 in each group. ^*^P < 0.05, ^**^P < 0.01 vs Control group; ^#^*P* < 0.05, ^##^*P* < 0.01 vs LPS group

We first examined whether stimulation with a 10 mg/kg dose of LPS for seven consecutive days promoted NE release from the myocardium of mice. As shown in Fig. 1a, myocardial NE content was significantly increased in LPS-challenged mice, which was not influenced by prazosin (PRA) at different doses (5, 10 and 20 mg/kg). Furthermore, the effect of PRA on cardiac function in LPS-stimulated mice was evaluated using echocardiography. The representative Doppler wave mode and M-mode echocardiograms of mice in each group were shown in Fig. 1b. The calculated results showed that continuous 10 mg/kg dose of LPS stimulation caused a slight reduction in left ventricular ejection fraction (EF) and fractional shortening (FS). The cardiac output (CO) and mitral E/A ratio of the mice in LPS group were significantly attenuated (Fig. 1c). PRA treatment at doses of 10 and 20 mg/kg significantly increased the levels of EF, FS, mitral E/A ratio, and CO in LPS-stimulated mice. However, the 20 mg/kg dose of PRA further reduced the systolic and diastolic blood pressure in the mice treated with LPS (Supplementary Fig. S1a). The systolic and diastolic blood pressure of the mice in the LPS + PRA (10 mg/kg dose) group were similar to those of control group. Therefore, this study adopted PRA at 10 mg/kg for the subsequent animal experiments. The ELISA result showed that a 10 mg/kg dose of PRA reduced the LPS-induced increase in the TNF-α in the heart of mice (Fig. 1d). Furthermore, the extent of myocardial fibrosis of the mice in each group was measured by Masson’s trichrome staining. As shown in Fig. 1e, more collagen deposition and larger fractional area of cardiac fibrosis were observed in the hearts of LPS-administered mice compared with that in the controls. The cardiac fibrosis of LPS-challenged mice was obviously relieved by treatment with PRA. The protein detection results revealed that the levels of α-SMA and collagen I/III, which were significant biomarkers of fibrosis, in the cardiac tissue of LPS-stimulated mice were markedly increased compared with that in control group. The LPS-induced elevation of α-SMA and collagen I/III was attenuated by PRA treatment (Fig. 1f). The Masson staining and Western Blot results showed that the α_1_-AR agonist, phenylephrine (PHE), exacerbated LPS-caused increase in collagen deposition and α-SMA expression (Fig. 1g-h). Quantifications of the relative protein levels were shown in Supplementary Fig. S1b. These data indicated that NE might promote LPS-associated myocardial fibrosis through activating α_1_-AR. PRA improved cardiac function, especially diastolic function, and alleviated myocardial fibrosis in the mice challenged with LPS. Activation of the α_1_-AR by PHE promoted myocardial fibrosis caused by LPS.

### The PKC-p38-Smad2/3 pathway activation and CHIP expression were regulated by α_1_-AR in the myocardium of mice with LPS injection

**Fig. 2:**
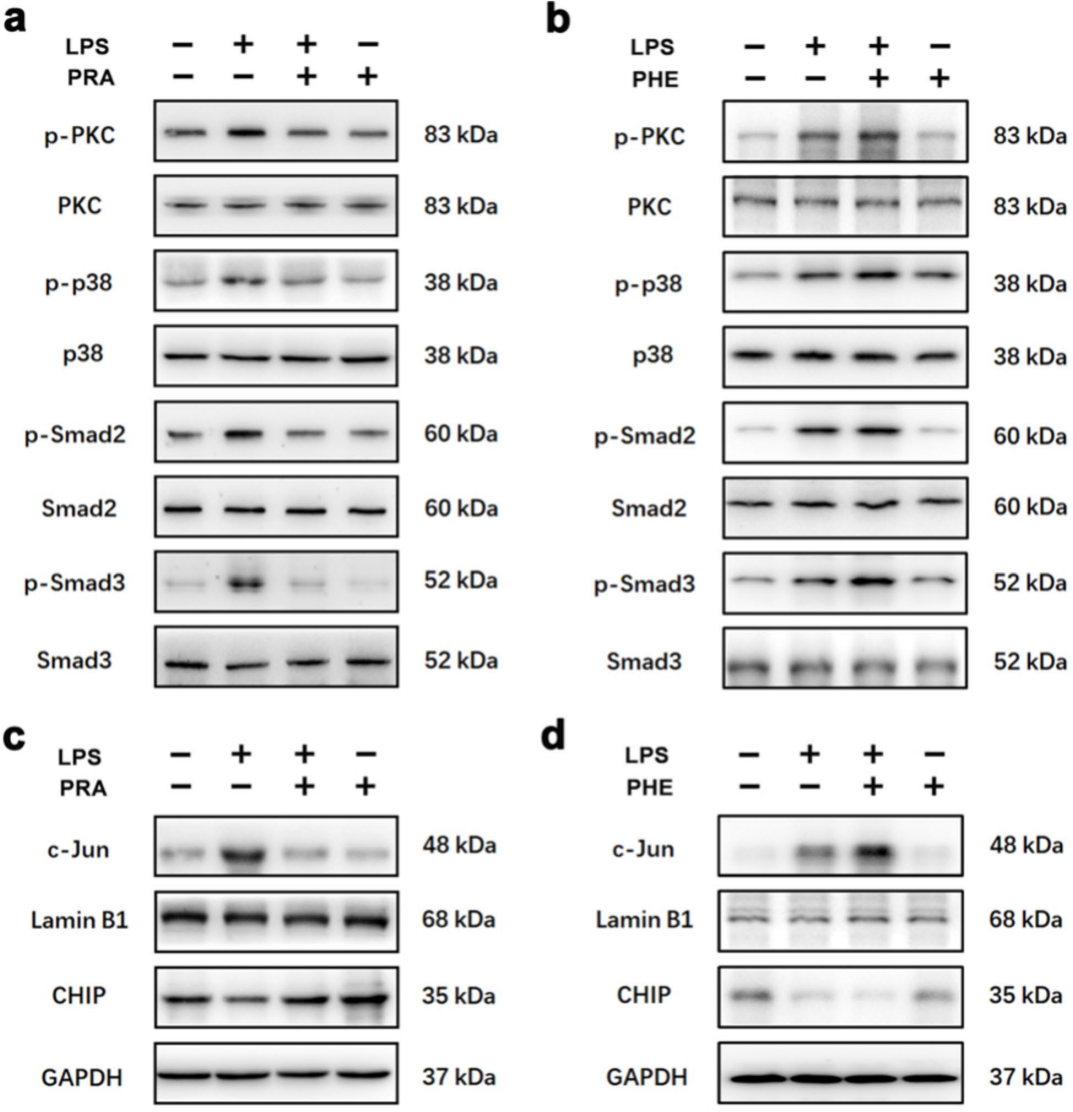
PRA and PHE regulated the PKC/p38 signaling pathway activation and CHIP expression in the heart of LPS-challenged mice. **a-b** The phosphorylation of PKC, p38 and Smad2/3 in the cardiac tissues of mice in each group was detected by Western Blot. **c-d** The levels of CHIP and nuclear c-Jun in the heart of mice were measured by Western Blot. *n* = 6 in each group

Previous research indicated that phosphorylation of PKC is subject to α_1_-AR activation in a number of cell types [31, 32]. To clarify the downstream pathway influenced by the antagonist or agonist of α_1_-AR with LPS, the phosphorylated PKC (p-PKC), p-p38 and p-Smad2/3 levels were examined by Western Blot. The results showed that the levels of p-PKC, p-p38, and p-Smad2/3 were significantly increased in the myocardium of mice stimulated by LPS, which were decreased by PRA treatment (Fig. 2a). In contrast, administration of PHE enhanced LPS-induced phosphorylation of PKC, p38, and Smad2/3 in the myocardial tissues of mice (Fig. 2b). Total PKC, p38 and Smad2/3 levels were unaffected by PRA/PHE with or without LPS stimulation. In addition to activating Smad2/3, phosphorylation of p38 also caused increased nuclear import of c-Jun [33], which inhibits CHIP expression as previously demonstrated in our study [30]. The Western Blot results proved that the nuclear c-Jun level was increased, and CHIP expression was decreased in myocardium of mice in the LPS group. PRA treatment reversed the nuclear c-Jun level and CHIP expression in the cardiac tissues of mice after LPS injection (Fig. 2c). PHE aggravated LPS-caused nuclear translocation of c-Jun and down-regulation of CHIP (Fig. 2d). The quantitative statistical results of Western Blot were shown in Supplementary Fig. S2a-b. These results suggested that antagonism of α_1_-AR by PRA not only inhibited PKC-p38-Smad2/3 signaling, but also restored CHIP expression by inhibiting c-Jun nuclear translocation.

### The effect of PRA against NE-mediated CFs differentiation was associated with attenuating activation of PKC and p38

**Fig. 3:**
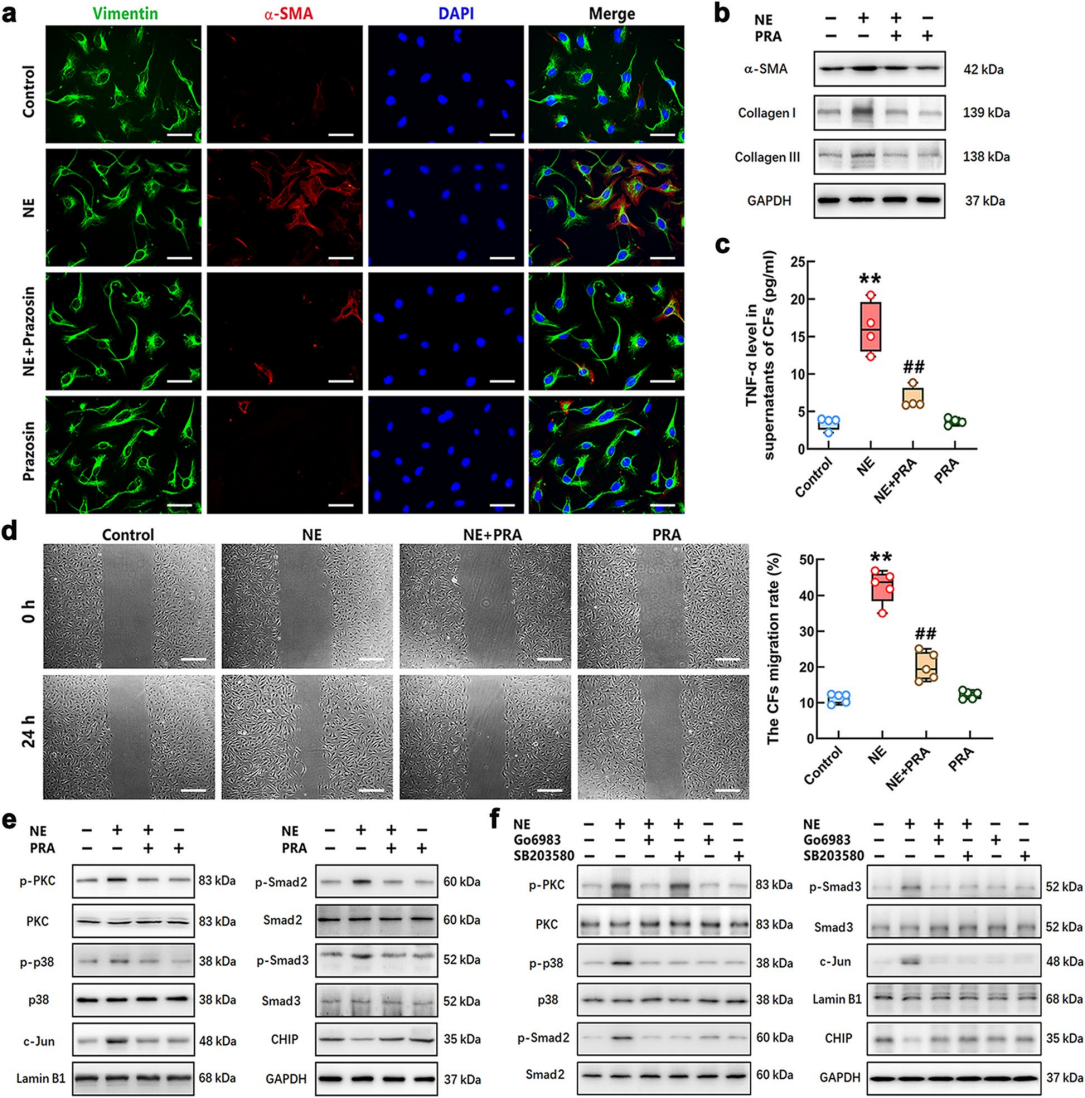
Blockage of α_1_-AR alleviated NE-mediated differentiation of CFs to myofibroblasts. **a** Representative immunofluorescence staining images of NE-stimulated CFs with or without PRA treatment. The CFs were stained with green (vimentin) and red (α-SMA) fluorescence. The nuclei were stained with DAPI (blue). Scale bar = 50 μm. **b** The α-SMA and collagen I/III levels were detected by Western Blot. **c** The content of TNF-α in CFs of each group was determined by ELISA. **d** The migratory ability of CFs was assessed using scratch assay (Scale bar = 200 μm.) and the quantification of the migration assay. **e–f** The phosphorylation of PKC, p38 and Smad2/3, and the levels of CHIP and nuclear c-Jun in the CFs of different groups were measured by Western Blot. *n* = 4–5 in each group. ^**^P < 0.01 vs Control group; ^##^*P* < 0.01 vs NE group

To determine the effect of PRA on the differentiation of CFs stimulated with NE, we detected the synthesis of α-SMA and collagen I/III in the CFs after PRA and NE treatment for 24 h. Both immunofluorescence (IF) and Western Blot results exhibited that the α-SMA levels in NE-stimulated cells were obviously increased compared with those in control cells. The levels of collagen I and III were markedly increased in CFs after NE stimulation. PRA restrained the NE-induced increase in α-SMA and collagen I/III in CFs (Fig. 3a-b). The ELISA result showed that TNF-α release of CFs after NE stimulation for 24 h was much higher than that in the control group, which was significantly decreased by PRA treatment (Fig. 3c). The conventional scratch healing assay was used to detect migratory capacity of CFs in this study. The results showed that NE reduced the cell-free scratch area, which was significantly restricted by PRA treatment (Fig. 3d). The Western Blot results proved that the levels of p-PKC, p-p38, p-Smad2/3 and the nuclear c-Jun were increased, and CHIP expression was reduced in CFs after NE stimulation. PRA treatment reversed this effect of NE on CFs (Fig. 3e). To further confirm the regulatory effect of the PKC/p38 axis on the NE-induced Smad2/3 and c-Jun activation, as well as downregulation of CHIP, we used inhibitors Go6983 and SB203580 for PKC and p38 to treat CFs 30 min prior to NE stimulation. The Western Blot results showed that both Go6983 and SB203580 reversed phosphorylated Smad2/3 and nuclear c-Jun level, and upregulated CHIP in NE-challenged CFs. Go6983 decreased NE-induced phosphorylation of p38 through inhibiting PKC activation (Fig. 3f). These quantitative statistical results of Western Blot were shown in Supplementary Fig. S3a-b. These data confirmed that NE promoted the differentiation of CFs to myofibroblasts by activating α_1_-AR, which might accelerate LPS-associated cardiac fibrosis. The suppressive effect of PRA on NE-induced CFs differentiation was related to the inhibition of PKC/p38-mediated Smad2/3 activation, and the upregulation of CHIP expression.

### CHIP overexpression inhibited NE-induced CFs differentiation and LPS-caused cardiac fibrosis by downregulating TGF-BR1 expression and Smad2/3 phosphorylation

**Fig. 4:**
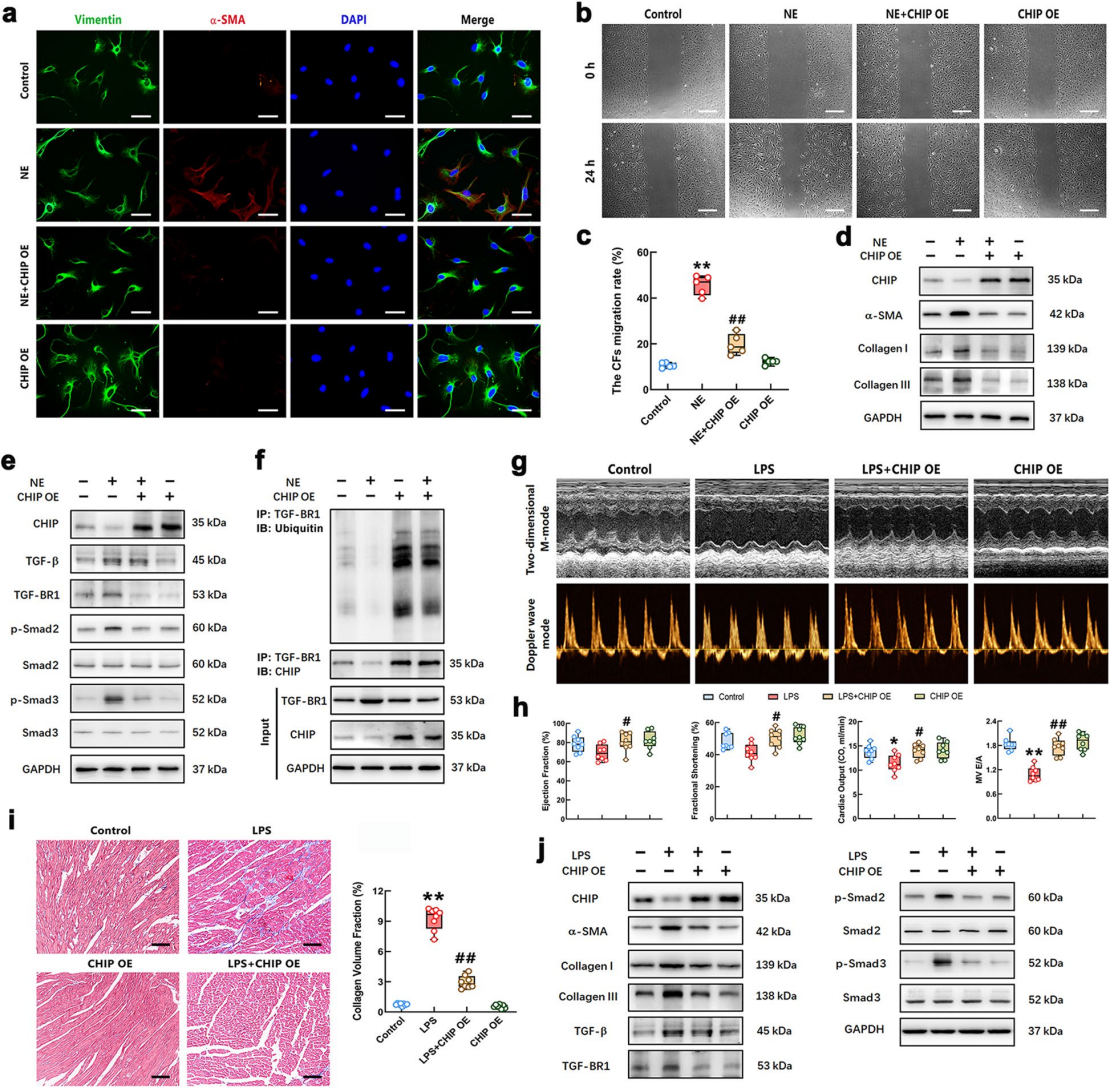
The effect of CHIP overexpression on NE-induced CFs differentiation and LPS-caused myocardial fibrosis. **a** The CFs were stained with green (vimentin) and red (α-SMA) fluorescence. Complete nuclei were stained with DAPI (blue). Scale bar = 50 μm. **b** The migration ability of CFs in each group was assessed using scratch assay (Scale bar = 200 μm). **c** Quantification of the migration assay. **d** The levels of α-SMA and collagen I/Ⅲ in the CFs of different group. **e** The contents of TGF-β, TGF-BR1, Smad2/3 and p-Smad2/3 in the CFs of different groups were measured by Western Blot. **f** Co-IP and Western Blot analysis for ubiquitination of TGF-BR1 and association between TGF-BR1 and CHIP in the CFs. **g** Representative echocardiograms of mice in each group. **h** Quantitative group data for echocardiographic analyses. **i** Representative Masson staining images showing interstitial collagen deposition in the cardiac tissues of mice (Scale bar = 100 μm) and quantification of the fractional fibrotic area in each group. **j** The levels of CHIP, α-SMA, collagen I/III, TGF-β, TGF-BR1, Smad2/3 and p-Smad2/3 in the heartof mice were measured by Western Blot. *n* = 4–8 in each group. ^*^P < 0.05, ^**^P < 0.01 vs Control group; ^#^*P* < 0.05, ^##^*P* < 0.01 vs NE group or LPS group

This study next sought to clarify the effect of CHIP on NE-induced CFs differentiation. As the results shown in Fig. 4a and 4d, CHIP overexpression (CHIP OE) significantly decreased the expressions of α-SMA and collagen I/III in CFs after NE stimulation. The result of the scratch test showed that the cell-free scratch area was obviously increased in the NE + CHIP OE group (Fig. 4b-c). The TGF-β-mediated TGF-BR1-Smad2/3 signaling pathway activation plays an integral role in cardiac fibrosis [34]. Here, we detected the expression of TGF-β, TGF-BR1, and p-Smad2/3 in the CFs challenged with CHIP OE and/or NE. The results showed that NE increased TGF-β level and slightly upregulated TGF-BR1 expression. CHIP overexpression obviously decreased the expression of TGF-BR1 and p-Smad2/3, but had no significant effect on the level of TGF-β (Fig. 4e). The quantitative statistical results of Western Blot were shown in Supplementary Fig. S4. We further detected the role of CHIP in the ubiquitination of TGF-BR1 and the interaction between TGF-BR1 and CHIP in CFs through Co-IP assay and Western Blot. The cells were treated with the proteasome inhibitor MG132 (10 μM) before harvesting to inhibit protein degradation. As shown in Fig. 4f, a decreased ubiquitination of TGF-BR1 was observed in CFs treated by NE. The up-regulation of CHIP markedly increased the level of ubiquitinated TGF-BR1 in CFs, and CHIP directly interacted with TGF-BR1 in CFs, which suggested CHIP down-regulated the expression of TGF-BR1 in CFs via ubiquitination. These results indicated that upregulation of CHIP suppressed NE-induced CFs differentiation through attenuating the expression of TGF-BR1, but not TGF-β, and inhibiting Smad2/3 phosphorylation.

We used the previously constructed heart-specific CHIP overexpression mice to further verify whether overexpression of cardiac CHIP could inhibit the myocardial fibrosis induced by LPS. The echocardiogram results showed that cardiac CHIP upregulation significantly increased the levels of EF, FS, mitral E/A ratio and CO in LPS-stimulated mice (Fig. 4g-h). The overexpression of CHIP markedly decreased LPS-induced collagen deposition and cardiac fibrosis in mice (Fig. 4i). The Western Blot result showed that the levels of α-SMA, collagen I/III, TGF-BR1 and p-Smad2/3 in myocardial tissue of LPS + CHIP OE group were significantly lower than in the LPS group, and TGF-β expression was not significantly different between the two groups (Fig. 4j), which confirmed that the effect of CHIP on restraining LPS-induced cardiac fibrosis was related to inhibiting TGF-BR1 expression and restraining the activation of Smad2/3.

### Downregulation of CHIP attenuated the effect of PRA on NE-triggered differentiation of CFs and cardiac fibrosis induced by LPS

**Fig. 5:**
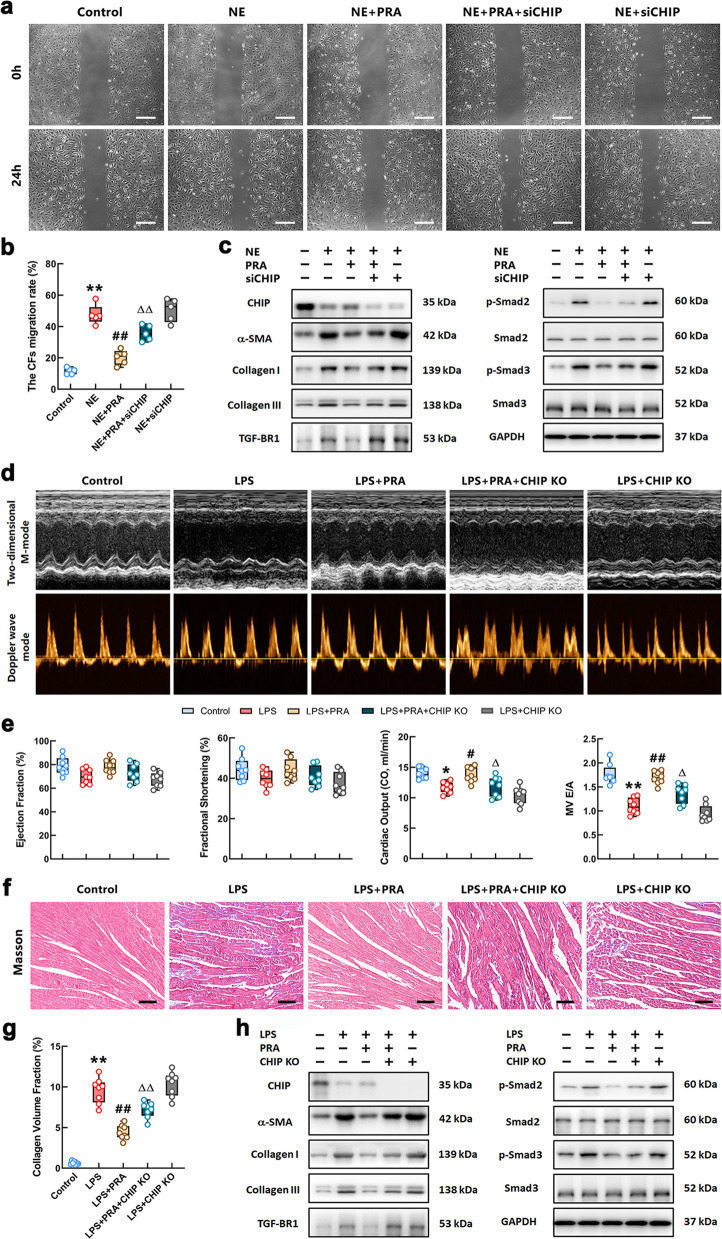
The anti-fibrotic effect of PRA was weakened by downregulation of CHIP in vitro and in vivo. **a** The migratory ability of CFs in each group was detected using scratch assay (Scale bar = 200 μm). **b** Quantification of the migration assay. **c** The levels of α-SMA, collagen I/III, TGF-BR1, total and p-Smad2/3 in the CFs of different groups were measured by Western Blot. **d** Representative echocardiograms of mice in each group. **e** Quantitative group data for echocardiographic analyses. **f** Representative Masson staining images showing interstitial collagen deposition in the myocardium of mice (Scale bar = 100 μm). **g** Quantification of the fractional area in the different groups. **h** The myocardial levels of CHIP, α-SMA, collagen I/III, TGF-β, TGF-BR1, Smad2/3 and p-Smad2/3 in the mice of different groups were measured by Western Blot. *n* = 5–8 in each group. ^*^P < 0.05, ^**^P < 0.01 vs Control group; ^#^*P* < 0.05, ^##^*P* < 0.01 vs NE group or LPS group; ^Δ^*P* < 0.05, ^ΔΔ^*P* < 0.01 versus NE + PRA + siCHIP group or LPS + PRA + CHIP KO group

We speculate that the anti-fibrotic effect of PRA is likely related to the upregulation of CHIP expression. To confirm this speculation, the CFs transfected with siRNA against CHIP (siCHIP) to knock down CHIP and the CHIP knockout (CHIP KO) mice were utilized in this study. The experimental result showed that knockdown of CHIP partially reversed the PRA-caused migration ability reduction of NE-challenged CFs (Fig. 5a-b). As the result shown in Fig. 5c, the suppressive effect of PRA on the expression of α-SMA, collagen I/III and TGF-BR1, as well as phosphorylation of Smad2/3 in NE-stimulated CFs was attenuated by CHIP knockdown. We further detected the influence of CHIP knockout in the protective effect of PRA on cardiac function of mice injected with LPS. As the results shown in Fig. 5d-e, the values ​​of EF and FS in CHIP KO mice were lower than those in wild type (WT) mice, but the difference between the two groups was not statistically significant. The effect of PRA on elevating CO and mitral E/A ratio of mice stimulated by LPS was attenuated by CHIP knockout. Moreover, the Masson staining result showed that knockout of CHIP alleviated the inhibitory effect of PRA on collagen deposition in the cardiac tissues of mice after LPS injection (Fig. 5f-g). Furthermore, the Western Blot result exhibited that CHIP knockout weakened the action of PRA on reducing the myocardial levels of α-SMA, collagen I/III, TGF-BR1 and p-Smad2/3 in the LPS-challenged mice (Fig. 5h). The quantitative statistical results of Western Blot were shown in Supplementary Fig. S5a-b. These findings further confirmed that the ability of PRA to restrain NE-induced CFs differentiation and LPS-caused myocardial fibrosis was associated with CHIP expression.

### LPS elevated the expression of the α_1A_-AR in the CFs

**Fig. 6:**
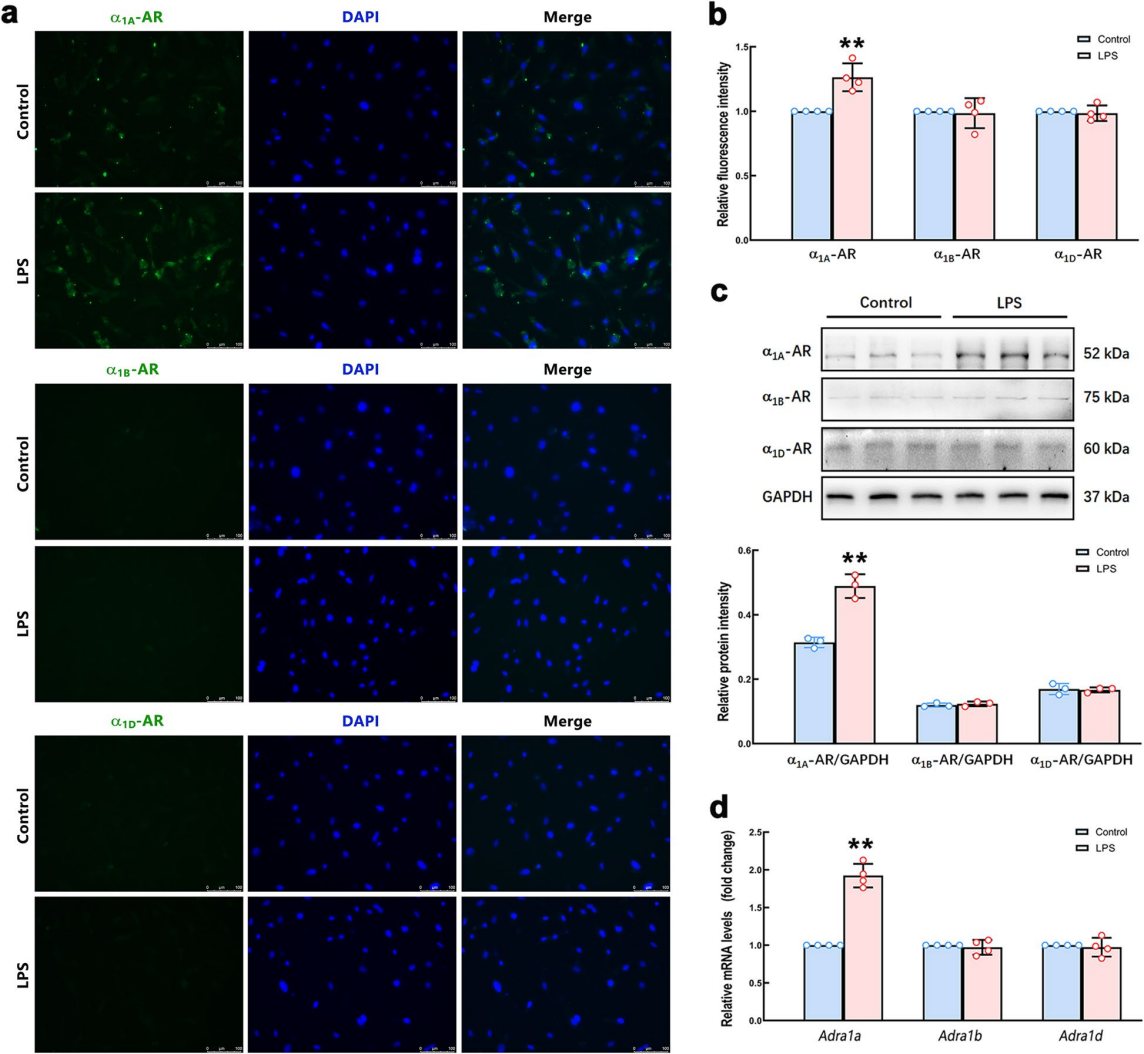
The expression of α_1A_-AR, α_1B_-AR and α_1D_-AR in CFs with or without LPS stimulation. **a** Representative immunofluorescence images of CFs with LPS or Saline stimulation for 24 h. The α_1A_-AR, α_1B_-AR and α_1D_-AR were labeled with green fluorescence, and DAPI-labeled nuclei showed blue fluorescence. Scale bar = 100 µm. **b** Quantification of the fluorescence intensity. **c** The protein levels of α_1A_-AR, α_1B_-AR and α_1D_-AR in CFs with or without LPS stimulation were detected by Western Blot. **d** The mRNA levels of α_1A_-AR (*Adra1a*), α_1B_-AR (*Adra1b*), and α_1D_-AR (*Adra1d*) in CFs of different groups were assayed by qRT-PCR. *n* = 3 or 4 in each group. ^**^P < 0.01 vs Control group

As a member of the G protein-coupled receptor family, α_1_-AR has been identified to have three subtypes, namely α_1A_-AR, α_1B_-AR and α_1D_-AR**.** This research further detected the protein and mRNA levels of α_1A_-AR, α_1B_-AR and α_1D_-AR in the CFs with and without LPS stimulation by IF, Western Blot and qRT-PCR. As the result shown in Fig. 6a-b, the intensity of green fluorescence representing α_1_-AR subtypes indicated that the α_1A_-AR expression was predominant on CFs. The expression of α_1A_-AR in LPS-stimulated CFs was higher than that in control cells. LPS had no effect on the expression of α_1B_-AR and α_1D_-AR. The levels of α_1A_-AR, α_1B_-AR and α_1D_-AR detected by Western Blot were consistent with the results of IF staining experiment (Fig. 6c). The qRT-PCR result showed that the mRNA level of α_1A_-AR was increased in the CFs after LPS stimulation. There was no significant difference in the mRNA contents of the α_1B_-AR and α_1D_-AR between the LPS and control groups (Fig. 6d). In addition, we found that neither NE nor CHIP had any effect on the expression of the three subtypes of α_1_-AR (Supplementary Fig. S6a). In the in vivo experiment, the results showed that LPS caused an increase in the expression of α_1A_-AR in the myocardium, but had no effect on the α_1B_-AR and α_1D_-AR expression. Neither PRA nor PHE affected this promoting effect of LPS on α_1A_-AR expression (Supplementary Fig. S6b). These results suggested that NE might cause the CFs differentiation by activating α_1A_-AR.

### Blockage of α_1A_-AR eliminated NE-mediated differentiation of CFs and LPS-triggered myocardial fibrosis

**Fig. 7:**
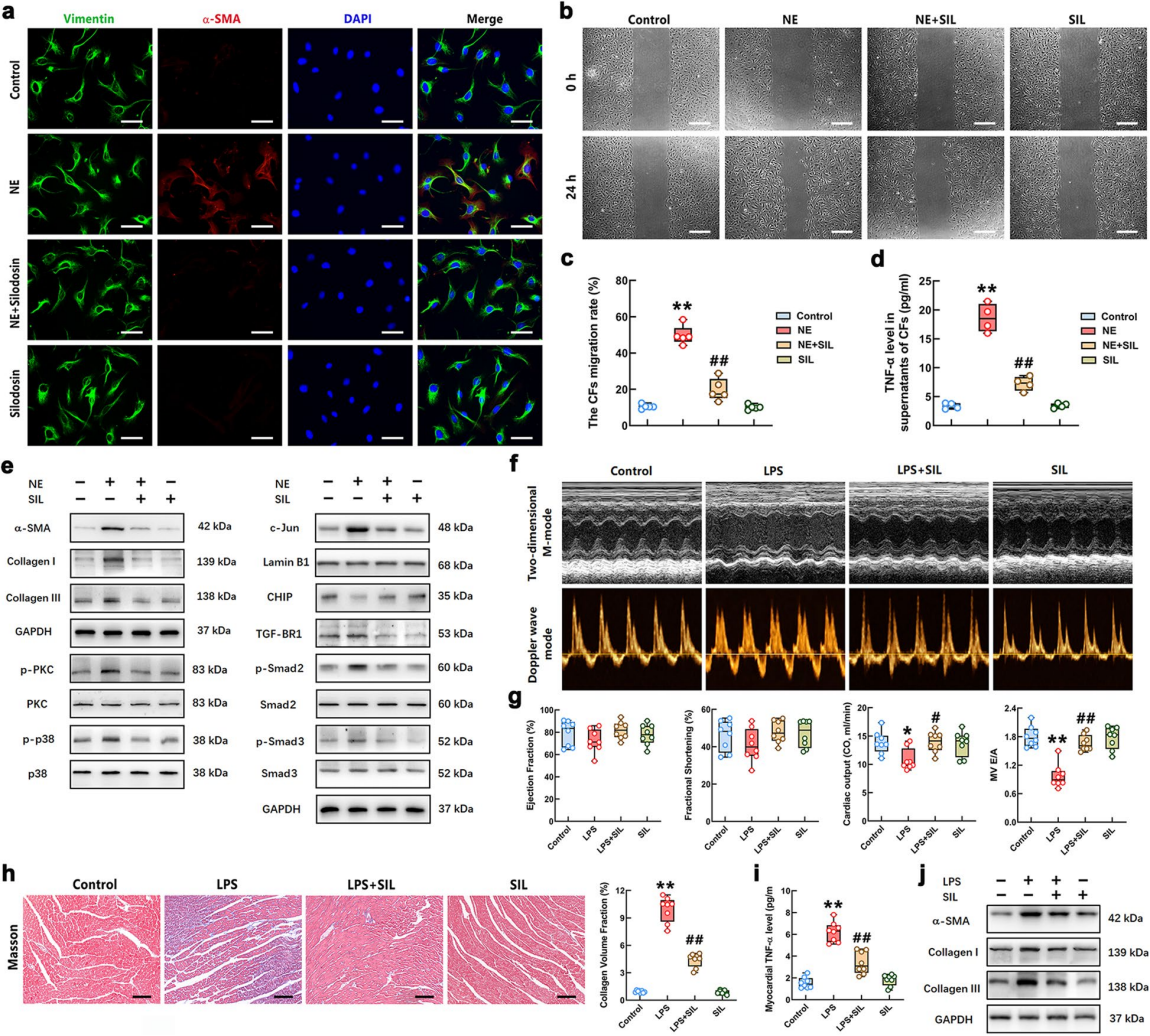
The effect of blocking α_1A_-AR on NE-simulated CFs differentiation and LPS-caused cardiac fibrosis. **a** The NE-challenged CFs with or without SIL treatment were labeled with green (vimentin) and red (α-SMA) and green fluorescence. Complete nuclei were stained with DAPI (blue). Scale bar = 50 μm. **b** The migratory ability of CFs in each group was assessed using scratch assay (Scale bar = 200 μm). **c** Quantification of the migration assay. **d** The level of TNF-α in CFs of each group was determined by ELISA. **e** The levels of α-SMA, collagen I/Ⅲ, PKC/p38 phosphorylation, nuclear c-Jun, CHIP, TGFBR1 and phosphorylation of Smad2/3 in the NE-stimulated CFs with or without SIL treatment were measured by Western Blot. **f** Representative echocardiograms of mice in each group. **g** Quantitative group data for echocardiographic analyses. **h** Representative Masson's trichrome staining images of myocardial sections (Scale bar = 100 μm.) and quantification of the fractional fibrotic area in different groups. **i** The TNF-α level of the cardiac tissues in the mice was measured by ELISA. **j** The protein levels of α-SMA and collagen I/III in the myocardium of mice were detected by Western Blot. *n* = 4–8 in each group. ^**^P < 0.01 vs Control group; ^##^*P* < 0.01 vs NE group or LPS group

This study further explored the role of a specific α_1A_-AR antagonist, silodosin (SIL), in NE-induced CFs differentiation. The IF result showed that SIL obviously reduced the expression of α-SMA after NE stimulation for 24 h (Fig. 7a). As the result shown in Fig. 7b-c, the effect of NE on enhancing the migration ability of CFs was markedly attenuated by SIL treatment. SIL treatment significantly decreased the TNF-α production of NE-stimulated CFs (Fig. 7d). Moreover, the Western Blot results showed that the levels of α-SMA, collagen I, collagen III, p-PKC, p-p38, p-Smad2/3 and the nuclear c-Jun were much lower, and CHIP expression was higher in the CFs of NE + SIL group compared with that in NE group (Fig. 7e). These results indicated that α_1A_-AR played a significant role in NE-caused differentiation of CFs. The inhibitory effect and molecular mechanism of the α_1A_-AR antagonist on CFs differentiation caused by NE were consistent with those of prazosin.

We further evaluated the effect of SIL on cardiac fibrosis and dysfunction in the mice after LPS administration. The results showed that SIL restored the levels of EF and FS, and significantly increased CO and E/A ratio in the mice after LPS stimulation (Fig. 7f-g). The Masson staining results showed that SIL decreased LPS-induced increase in volume fraction of collagen (Fig. 7h). As shown in Fig. 7i, SIL inhibited the upregulation of myocardial TNF-α induced by LPS. Moreover, SIL significantly reduced the contents of α-SMA and collagen I/III in the cardiac tissues of mice after LPS stimulation (Fig. 7j). The quantitative statistical results of Western Blot were shown in Supplementary Fig. S7. These results suggested that the blockage of α_1A_-AR inhibited LPS-caused cardiac fibrosis and dysfunction in the mice.

## Discussion

Numerous clinical studies have indicated that myocardial fibrosis in septic patients with significant cardiac dysfunction adversely affects the prognosis [35, 36]. Although it is generally considered that excessive inflammatory response caused by LPS during sepsis is a crucial cause of myocardial fibrosis, the mechanisms underlying sepsis-related cardiac fibrosis are not fully elucidated. In this study, we found that the content of NE was increased in the myocardium of LPS-stimulated mice, and NE facilitated the differentiation of CFs to myofibroblasts, which was abrogated by both the α_1_-AR and α_1A_-AR antagonists, prazosin and silodosin. The predominant subtype of α_1_-AR expressed on cardiac fibroblasts was α_1A_-AR, which was upregulated by LPS stimulation. These critical and innovative findings provide an essential basis for the establishment of a new effective therapeutic strategy to improve myocardial fibrosis and survival in patients with sepsis.

This study established a myocardial fibrosis model by injecting mice with low-dose LPS intraperitoneally for seven consecutive days. We found that the cardiac function of mice, especially the diastolic function and cardiac output, was significantly reduced after LPS stimulation. Meanwhile, NE, myocardial fibrosis and collagen deposition levels were markedly elevated in the LPS-injected mice. Although LPS-induced inflammation has a vital effect on septic cardiac fibrosis, NE is also one of the reasons that should be noticeable. The studies have proved that increased circulating NE were involved in the molecular mechanism of pulmonary remodeling [37, 38]; the sympathetic system and NE play an important role in liver fibrosis through acting on stellate cells [39]. It has been demonstrated that NE stimulation increased cytosolic calcium and collagen I/III in CFs [40]. This study confirmed that cardiac NE content and biomarker proteins of myocardial fibrosis in mice were enhanced after LPS stimulation, indicating that increased NE production aggravated cardiac fibrosis. Prazosin, as a selective α_1_-AR antagonist, directly blocks the binding of NE to α_1_-AR and is often used to treat clinical diseases such as hypertension and benign prostatic hyperplasia [41]. The current study confirmed that prazosin improved myocardial fibrosis and cardiac function in LPS-stimulated mice. On the contrary, the α_1_-AR agonist, phenylephrine, aggravated myocardial fibrosis caused by LPS. We further examined the effect of prazosin on NE-caused differentiation of CFs to myofibroblasts. The results of in vitro experiments showed that prazosin obviously attenuated the NE-induced differentiation of CFs, suggesting that NE exerts this effect mainly through the activation of α_1_-AR on CFs.

The activation of α_1_-AR induces PKC phosphorylation through diglyceride, and Ca^2+^ synergizes with phosphatidylserine, further activating p38 kinase [42]. Several studies have demonstrated that PKC inhibitors restrained the phosphorylation of p38 in both in vitro and in vivo studies [43, 44]. The development of cardiac fibrosis is closely related to the p38 and Smad2/3 pathway activation. Kojonazarov B et al. reported that the phosphorylation of p38 promotes TGF-β-mediated differentiation of cardiac fibroblasts to myofibroblasts through activating Smad2/3 [45]. Sacubitril and valsartan attenuated angiotensin II-induced atrial fibrosis by inhibiting the phosphorylation of p38 and Smad2/3 [46]. This study also confirmed the regulatory role of PKC/p38 in activation of Smad2/3 by using the inhibitors. Both PKC and p38 inhibitors attenuated NE-induced Smad2/3 activation and blockage of PKC restrained p38 phosphorylation in the CFs stimulated by NE. Therefore, it is speculated that NE may activate the PKC-p38-Smad2/3 axis by binding with α_1_-AR, leading to the differentiation of cardiac fibroblasts, which was prevented by the action of prazosin. The data in this study supported that prazosin inhibited the phosphorylation of PKC, p38, and Smad2/3 in the myocardium of mice injected with LPS and in NE-stimulated cardiac fibroblasts, demonstrating that the inhibitory effect of prazosin on NE-mediated cardiac fibroblasts differentiation was related to the blocking of PKC-p38-Smad2/3 signaling pathway, thereby alleviating LPS-induced myocardial fibrosis.

Our previous study reported that the expression of E3 ligase CHIP in cardiac tissue is decreased during sepsis, and the activation of transcription factor c-Jun induced by LPS is an important reason for the down-regulation of CHIP expression [30]. The nuclear translocation of c-Jun, which subsequently initiates the expression of target genes, is closely related to the activation of p38 [47]. It has been demonstrated that inhibition of p38 obviously suppressed c-Jun-mediated signaling pathways in animal studies and cell experiments [48, 49]. We demonstrated that the inhibitors of PKC and p38 restrained NE-induced nuclear translocation of c-Jun. Thus, it is assumed that the PKC/p38 phosphorylation induced by NE not only activated Smad2/3, but also downregulated the expression of CHIP through activating c-Jun. Prazosin may relieve the influence of NE on CHIP and restore its expression in CFs. The results of the present study confirmed that prazosin significantly reduced the level of nuclear c-Jun and recovered the expression of CHIP in the myocardium of LPS-stimulated mice and the NE-challenged cardiac fibroblasts. CHIP is highly expressed in cardiac cells and improves cardiac damage caused by various damage factors [28, 50]. In this study, we found that CHIP overexpression in CFs inhibits NE-induced differentiation of cells. Meanwhile, the LPS-induced myocardial fibrosis was restrained in cardiac-specific CHIP-overexpressing mice. It has been found that overexpression of CHIP downregulated the level of Smad3 through the ubiquitin-dependent degradation pathway, thereby desensitizing cells to TGF-β signaling [29, 51]. In the current study, we deeply investigated whether CHIP regulated the expressions of TGF-β and TGF-BR1, the upstream signaling proteins of Smad2/3. Interestingly, we found that upregulation of CHIP suppressed TGF-BR1 expression in the CFs after NE stimulation, but not TGF-β. CHIP directly interacted with TGF-BR1 and promoted its ubiquitination modification, which indicated that TGF-BR1 was likely to be a potential substrate of CHIP. Our study innovatively identified an additional regulatory role of CHIP in suppressing Smad2/3 activation by down-regulating TGF-BR1 expression. In addition, by using transgenic mice with CHIP knockout, we found that the knockout of CHIP weakened the effect of prazosin on alleviating LPS-caused myocardial fibrosis and inhibiting the activation of related signaling pathways. This study confirmed that prazosin reversed the inhibition of NE on CHIP expression by blocking α_1_-AR, which helped to further constrain NE-mediated Smad2/3 activation and cardiac fibroblast differentiation, leading to preventing myocardial fibrosis caused by LPS.

As a member of the G protein-coupled receptor family, α_1_-AR has been identified to have three subtypes, namely α_1A_-AR, α_1B_-AR and α_1D_-AR [52, 53]. Some previous studies have suggested that, in the rodent heart, cardiomyocytes express only the α_1A_-AR and α_1B_-AR subtypes [54], with α_1B_-AR more abundant than α_1A_-AR, whereas the α_1D_-AR is located in coronary arteries [55, 56]. The three subtypes of α_1_-AR were not expressed in rodent cardiac fibroblasts at all, and thus were uninvolved in the cardiac fibroblast proliferation that characterizes maladaptive remodeling [57]. In this study, it was found that α_1A_-AR, α_1B_-AR and α_1D_-AR were present in cardiac fibroblasts. The mRNA and protein levels of all three α_1_-AR subtypes were detected by qPCR, IF staining and Western Blot in this research. Surprisingly, all three isoforms of α_1_-AR were expressed on the CFs, and the expression of α_1A_-AR was dominant and much higher than the other two subtypes. Moreover, we observed that the expression of α_1A_-AR in CFs was increased in response to LPS stimulation. Therefore, we speculate that NE causes the differentiation of cardiac fibroblasts mainly by activating α_1A_-AR, which is confirmed by the experimental results of the present study. The α_1A_-AR antagonist, silodosin, was able to inhibit NE-induced differentiation of CFs to myofibroblasts and reduce the α-SMA and collagen I/III production. This effect of silodosin was associated with the inhibition of PKC-p38-Smad2/3 signaling pathway, and upregulation of CHIP level, which attenuated TGF-BR1 expression and suppressed Smad2/3 activation. Just as we expected, silodosin also ameliorated myocardial fibrosis and cardiac function in LPS-stimulated mice. These results confirmed that the activation of α_1A_-AR played a main role in NE-induced differentiation of cardiac fibroblasts, which promoted myocardial fibrosis in mice with LPS injection. Since the cardiac tissue includes cardiomyocytes and endothelial cells, in addition to fibroblast, the blockage of α_1B_-AR in cardiomyocytes and α_1D_-AR in endothelial cells by prazosin may have an uncertain influence on the biological functions of these two cell types. Silodosin is the antagonist with a specific affinity for α_1A_-AR and has been recommended as a clinical medicine because of its safety, efficacy, and good tolerability [58, 59]. Therefore, blockage of α_1A_-AR by silodosin could more precisely inhibit NE-induced differentiation of cardiac fibroblasts without affecting other cells in the heart.

The current study still has limitations. Firstly, although we have revealed the molecular mechanism by which blockage of α_1A_-AR restrained the cardiac fibroblasts differentiation induced by NE, there may still exist other α_1A_-AR-mediated downstream pathways that are related to fibrosis. We are currently conducting experiments to explore other proteins and potential pathways associated with α_1A_-AR. Secondly, the animal model of sepsis established through the cecal ligation and puncture (CLP) surgery more realistically simulates sepsis caused by peritoneal infection in clinic. Although the chronic LPS stimulation is conducive to establishing a myocardial fibrosis model that mimics sepsis, to better align with the clinical situation, the therapeutic effect of α_1A_-AR antagonist on cardiac fibrosis in a CLP-related animal model needs to be investigated in the future. Thirdly, in the early or mild stage of sepsis, blood pressure usually remains normal. However, as the condition progresses, blood pressure may continue to decline. Since the α_1A_-AR exists in the peripheral vascular smooth muscle, the influence of silodosin on blood pressure in septic model still needs to be further evaluated to clarify the safety of its usage. Nevertheless, this study still provided novel perspectives on the pathogenesis of LPS-associated cardiac fibrosis and new insights for the future research on septic cardiac dysfunction. These findings revealed the innovative effect and molecular mechanism of α_1_-AR blockage on inhibiting LPS-induced myocardial fibrosis.

**Fig. 8:**
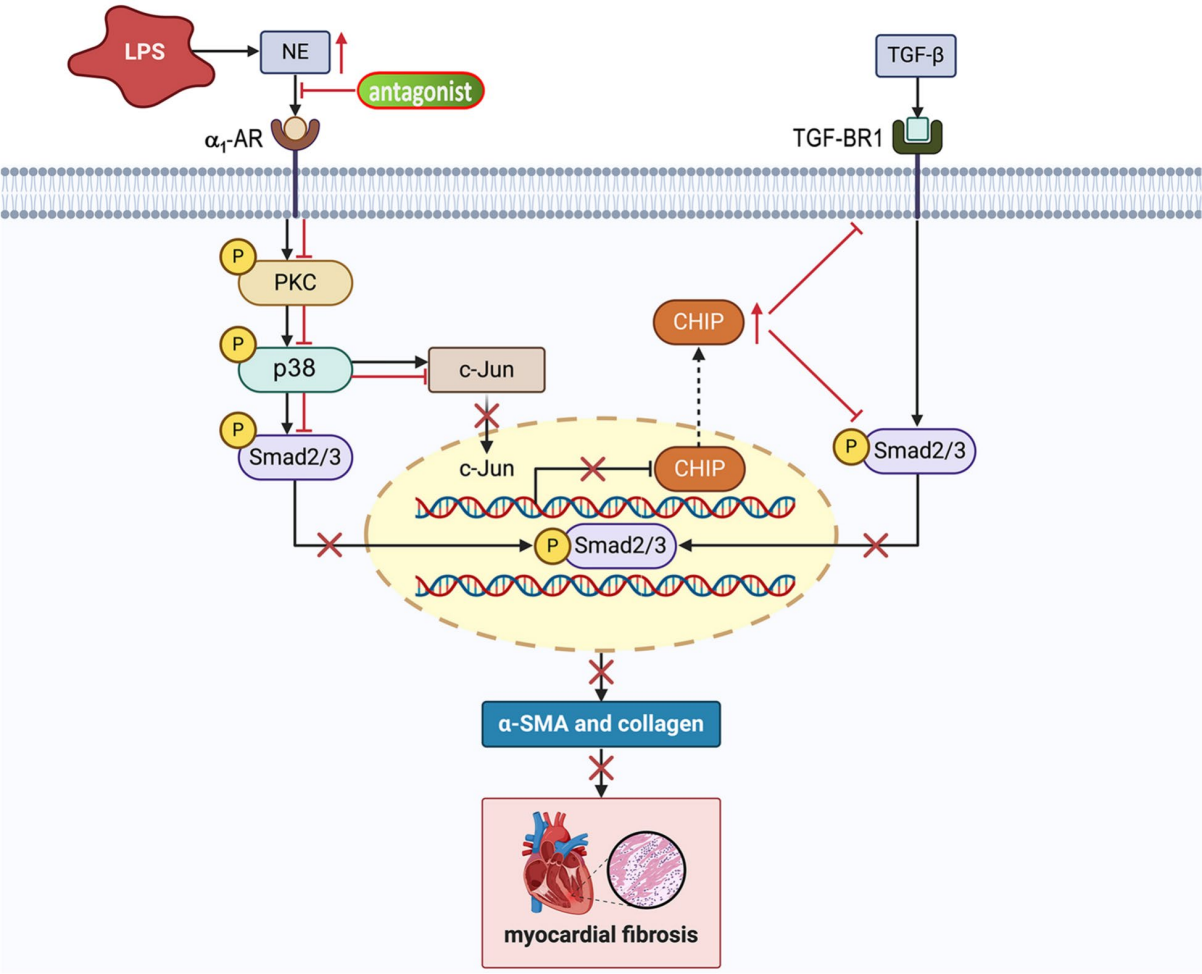
Molecular mechanism of blocking α_1_-AR on attenuating LPS-induced cardiac fibrosis. Blockage of α_1_-AR not only inhibits NE-mediated activation of PKC-p38-Smad2/3 signaling pathway; but also prevents c-Jun from entering the nucleus, which restores the level of CHIP, resulting in downregulation of TGF-BR1 and further suppression of Smad2/3. By means of these effects, the blockade of α_1_-AR reduces NE-induced differentiation of cardiac fibroblasts to myofibroblasts, resulting in attenuating LPS-induced myocardial fibrosis. The BioRender was used to creat this figure (Agreement number: NN28YJNY6W)

In our research, LPS induces NE release in the myocardial tissue, accelerating myocardial fibroblast differentiation through activating α_1_-AR. We provide the first evidence that the α_1A_-AR is the major subtype of α_1_-AR on CFs. The suppressive effect of α_1_-AR blockage on NE-induced differentiation of cardiac fibroblasts is associated with restraining PKC-p38-Smad2/3 signaling pathway, and restoring CHIP expression that downregulates TGF-BR1 and further suppresses Smad2/3 activation, resulting in alleviating LPS-caused myocardial fibrosis (Fig. 8). This study reveals new pharmacological effects and potential application value of prazosin and silodosin, and provides an important experimental basis for the establishment of a new therapeutic strategy to improve myocardial fibrosis and cardiac function in septic patients.

## Materials and methods

A list of the primary reagents and antibodies used in this research is provided in the Table S1 of Supplementary material.

### Experimental animals and treatment procedures

Wild-type male C57BL/6 mice, cardiac-specific CHIP overexpressing mice and CHIP knockout (CHIP^−/−^) mice aged 8 to 10 weeks were used in this research. Wild-type male C57BL/6 mice were purchased from Guangdong Experimental Animal Center. The CHIP knockout (CHIP^−/−^) mice were obtained from Shanghai Model Organisms (Shanghai, China). Cardiac-specific CHIP overexpression mice were obtained as previously described [30]. All the mice used in the experiments were from the same generation.

The mice were intraperitoneally injected with LPS (10 mg/kg) daily for one week. The mice treated with normal saline instead of LPS were served as controls. The mice were injected subcutaneously with PRA (1 mg/kg body weight), or PHE (50 μg/kg body weight), or SIL (0.1 mg/kg body weight) after LPS injection for 1 h. Two hours after the last LPS injection, mice were anesthetized with isoflurane for ultrasound testing and then executed to obtain the left ventricle for subsequent assays.

### Echocardiographic analyses and tail artery blood pressure measurement

After seven consecutive days of LPS injections, under 2% isoflurane anesthesia, transthoracic 2-dimensional M-mode imaging from the parasternal short-axis and spectral Doppler of cardiac aortic and mitral valve flow were acquired using a VINNO6LAB small animal ultrasound imaging system (Feiyinuo Technology Co., Ltd.). The EF, FS, CO, and mitral E/A ratio were calculated by the software of the imaging system, and the data were averaged based on three consecutive cardiac cycles at least. The systolic and diastolic blood pressure of mouse tail artery were recorded using the noninvasive CODA tail-cuff system (Kent Scientific Corporation, Torrington, CT, USA).

### Masson’s trichrome stain

The left ventricle was fixed in 4% paraformaldehyde for 24 h. After fixation, the tissue was embedded in paraffin and sectioned at a thickness of 5 μm. The paraffin-embedded tissue sections were stained using Masson’s trichrome staining per the manufacturer's protocol and then assessed using a light microscope. The fractional area (collagen area/total area) of cardiac fibrosis was quantified using image J software.

### Cardiac fibroblast isolation and treatment

The CFs were isolated from adult male C57BL/6 mice. Briefly, the ventricles were minced and digested at 37 °C using 0.1% collagenase II and 0.125% trypsin in a five-minute cycle. The cell suspension from each digestion was filtered through a 70-µm cell strainer and then centrifuged. The cells pellets were resuspended using DMEM containing 10% FBS and inoculated at a density of 1.0 × 10^5^ cells per well in 6-well plates for 2 h at 37 °C with 5% CO_2_. The cells were given a fluid change, and the remaining adherent cells were fibroblasts. Then, after 48 h of culture, fibroblasts were treated with PRA (5 μM) or SIL (10 μM) for 30 min, followed by treatment with saline or NE (0.2 µM). In separate experiments, CFs were pre-incubated with SB203580 (1 µM) or Go6983 (1 µM) for 30 min before NE stimulation.

### Plasmid transfection

The pLenti-CHIP plasmid constructed for CHIP expression was extracted using the Plasmid Midi Kit (QIAGEN). Short interfering RNA (siRNA) specific to mouse CHIP and a control siRNA were obtained from RIBOBIO (Guangdong, China). The siRNA sequence used to knock down CHIP is 5’-GCTAGAGATGGAGAGTTAT-3’. After 24 h of fibroblast adherence, the plasmids were transfected into cells with Lipofectamine 3000 reagent according to the manufacturer's instructions. The efficiency of transfection was analyzed by Western Blot.

### Enzyme-linked immunosorbent assay (ELISA)

For in vivo experiments, the left ventricle was homogenized thoroughly in saline and then centrifuged at 13,000 × rpm for 15 min at 4 °C, and the supernatant was harvested. For in vitro experiments, the cells were stimulated with NE for 24 h, and the supernatants were harvested. The levels of TNF-α were detected by using an ELISA kit according to the manufacturer's instructions.

### Immunofluorescence staining

Primary mouse CFs were fixed with 4% paraformaldehyde for 15 min, and then treated with 0.1% Triton X-100. After washing three times, the cells were covered with blocking solution and incubated for 1 h at room temperature. After discarded blocking solution, the CFs were incubated with antibodies against α-SMA and vimentin at a dilution of 1:200 at 4 °C for overnight. Then, the CFs were incubated with the anti-mouse secondary antibody labeled with Alexa Fluor 555 for α-SMA and anti-rabbit secondary antibody labeled with Alexa Fluor 488 for vimentin (1:400 dilution) at room temperature for 1 h. Finally, the nucleus was stained with DAPI (1:200 dilution). The fluorescence-labeled CFs were observed using a Leica fluorescence microscope.

### Co-immunoprecipitation (Co-IP) assays

The CFs with or without CHIP overexpression were washed with ice-cold PBS after NE stimulation for 4 h and then lysed with RIPA lysis buffer. The supernatant was incubated with 2 μg of anti-TGF-BR1 antibody for 1 h at 4 °C. Subsequently, the protein A/G beads were added and incubated for overnight at 4 °C. After three washes, the Co-IP samples were processed in loading buffer under reducing condition and protein binding was detected by Western Blot.

### Western blot assay

The RIPA buffer containing PMSF was used to extract total proteins. Nuclear proteins were extracted using a nuclear protein extraction kit, following the manufacturer's instructions. Proteins were separated by SDS-PAGE (sodium dodecyl sulfate–polyacrylamide gel electrophoresis), and then transferred to polyvinylidene difluoride (PVDF) membranes. After blocking with 5% skim milk, the PVDF membranes were incubated with the appropriate primary antibodies (1:1000 dilution) overnight at 4 °C. Following incubation with a horseradish peroxidase (HRP)-coupled secondary antibody (1:20,000 dilution) at room temperature for 1 h and extensive washing three times, the immunoblotted proteins were visualized using an enhanced chemiluminescence reagents. Image J software is used to quantify the blots.

### Quantitative real-time polymerase chain reaction (qRT-PCR)

Total RNA was isolated from CFs using TRIzol reagent and then reverse-transcribed into cDNA using a reverse transcription kit. Subsequently, the cDNA was amplified using the ChemoHS-qPCR mix and the signals were detected by CFX Maestro software v1.1 (Bio-Rad, Hercules, CA, USA). Finally, the relative expressions of genes were determined using the 2(^−ΔΔct^) method normalized to an internal control (*Gapdh*). The primer sequences for qRT-PCR are shown in Supplemental Table S2.

### Migration assay

The CFs were inoculated at a density of 5 × 10^5^ cells per well in 6-well plates. After 24 h, CFs were cultured in FBS-free DMEM and pretreated with PRA or SIL for 30 min. Subsequently, the CFs were treated with saline or NE and the cell monolayer was scraped with a sterile pipette tip. The scratched area was photographed using an IX51 inverted phase contrast fluorescence microscope at 0 h and 24 h after scratching and quantified by Image J software. The relative cell migratory rate was calculated according to the formula: (width at 0 h—width at 24 h)/width at 0 h.

### Statistical analysis

All quantitative data were expressed as mean ± standard error of the mean (SEM), Statistical significance between two groups was analyzed by using Student’s t-test of two independent samples, and differences among multiple groups were determined using one-way ANOVA followed by Dunnett’s, Tukey’s, or Bonferroni's test as appropriate. *P* < 0.05 was considered statistically significant. SPSS software were used for statistical analyses of experimental data.

## Supplementary Information

Supplementary Material 1.

## Data Availability

All the datasets supporting this study are presented in the article/supplemental material and will be made available upon reasonable request.
