# Supplementary material for: E3 ligase CHIP restoration facilitates the effect of α1-adrenoceptor blockage on alleviating lipopolysaccharide-caused cardiac fibrosis via downregulating TGF-BR1 expression and Smad2/3 activation
Source: Mol Biomed. 2025 Nov 10;6:106. doi: 10.1186/s43556-025-00357-5 (PMC12602763; doi:10.1186/s43556-025-00357-5)
Supplement: Supplementary file 1 — Supplementary Material 1. [file 43556_2025_357_MOESM1_ESM.docx]

**Supplementary Information**

**E3 ligase CHIP restoration facilitates the effect of α_1_-adrenoceptor blockage on alleviating lipopolysaccharide-caused cardiac fibrosis via downregulating TGF-BR1 expression and Smad2/3 activation**

Wan Lin^1*^, Hang Li^1*^, Hin Fong^1*^, Xingyu Su^1^, Junhao Wen^1^, Xianyun shao^1^, Ziqing Yan^1^, Yiyang Wang^1#^

1. Department of Pathophysiology, School of Medicine, Jinan University, Guangzhou 510632, Guangdong, China.

*These authors contributed equally to this work.

^#^Corresponding author: Yiyang Wang, wangyiyang@jnu.edu.cn

**Table S1. Major resources**

| **Antibodies** | **Cat Number** | **Supplier/Vendor** |
| --- | --- | --- |
| anti-GAPDH | #2118S | Cell Signaling Technology |
| anti-CHIP | #C3B6 | Cell Signaling Technology |
| anti-c-Jun | #9165S | Cell Signaling Technology |
| anti-LaminB1 | #13435S | Cell Signaling Technology |
| anti-p-p38 | #4511S | Cell Signaling Technology |
| anti-p38 | #9212S | Cell Signaling Technology |
| anti-TGF-β | #3711S | Cell Signaling Technology |
| anti-Vimentin | #5741S | Cell Signaling Technology |
| anti-p-PKC | #13618 | Signalway Antibody |
| anti-PKC | #41347 | Signalway Antibody |
| anti-p-Smad2 | #3108 | Cell Signaling Technology |
| anti-Smad2 | # 5339 | Cell Signaling Technology |
| anti-p-Smad3 | # 9520 | Cell Signaling Technology |
| anti-Smad3 | #9523 | Cell Signaling Technology |
| anti-collagen Ⅰ | WL0088 | Wanleibio |
| anti-collagen Ⅲ | WL03186 | Wanleibio |
| anti-TGF-BR1 | WL03150 | Wanleibio |
| anti-TGF-BR1 | sc-518086 | Santa Cruz Biotechnology |
| anti-α-SMA | ab7817 | abcam |
| anti-α_1A_-AR | sc-100291 | Santa Cruz Biotechnology |
| anti-α_1B_-AR | AF8537 | R&D Systems |
| anti-α_1D_-AR | sc-390884 | Santa Cruz Biotechnology |
| anti-ubquitin | sc-8017 | Santa Cruz Biotechnology |
| Donkey anti-rabbit 488 | A21206 | Invitrogen |
| Donkey anti-mouse 555 | A31570 | Invitrogen |
| Goat anti-Rabbit IgG (H+L) Secondary An-tibody, HRP | 31460 | Thermo Fisher Scientific |
| Goat anti-Mouse IgG (H+L) Secondary An-tibody, HRP | 31430 | Thermo Fisher Scientific |
| Protein A/G PLUS-Agarose | sc-2003 | Santa Cruz Biotechnology |
| Agents |  |  |
| LPS | Escherichia coli, 055: B5 | Sigma Aldrich |
| Norepinephrine | HY-13715A | MedChemExpress |
| Prazosin | HY-B0193A | MedChemExpress |
| Silodosin | HY-10122 | MedChemExpress |
| Tamoxifen | HY-13757A | MedChemExpress |
| Go6983 | HY-13689 | MedChemExpress |
| SB203580 | HY-10256 | MedChemExpress |
| QRT-PCR |  |  |
| RNA extraction reagent | #9109 | TaKaRa |
| Trizol | AG21101 | Accurate Biology |
| Reverse Transcription Kit | AG11711 | Accurate Biology |
| ChemoHS Q-PCR mix | MQ00401 | Monad |
| Miscellaneous |  |  |
| DAPI solution | #D523 | Dojindo Molecular Technologies |
| Plasmid Midi Kit | 12143 | QIAGEN |
| Lipofectamine®3000 Transfection Reagent | L3000001 | Thermo Fisher Scientific |
| Fetal Bovine Serum | FBSCN500-S | AusGeneX |
| Type Ⅱ collagenase | V900892 | Sigma Aldrich |
| 2.5％ Trypsin | Gibco | 15090-046 |
| TNF-α ELASA kit | MTA00B | R&D Systems |
| Nuclear and Cytoplasmic  Extraction Reagents | 78833 | Thermo Fisher Scientific |
| Masson trichrome stain kit | C0189S | Beyotime |

**Table S2. Primer sequences used for QRT-PCR assay**

| **Gene (Protein)** | **Forward Sequence** | **Reverse Sequence** |
| --- | --- | --- |
| *Gapdh* (GAPDH) | TGAGCCTCCTCCAATTCAACC | TCACACCGACCTTCACCATT |
| *Adra1a* (α_1A_-AR) | AAGTTCCGAGCCGCTCTTTT | TCGCTTCTTGCTTCTCCTGG |
| *Adra1b* (α_1B_-AR) | CTGGTCTTAGCTTCGTGGCA | GTCAAGCCATGGGTTCAGAGA |
| *Adra1d* (α_1D_-AR) | GCTGCTCCCAGGGAAAAGAT | ACAGATTTGTATGGCGGGCA |

**
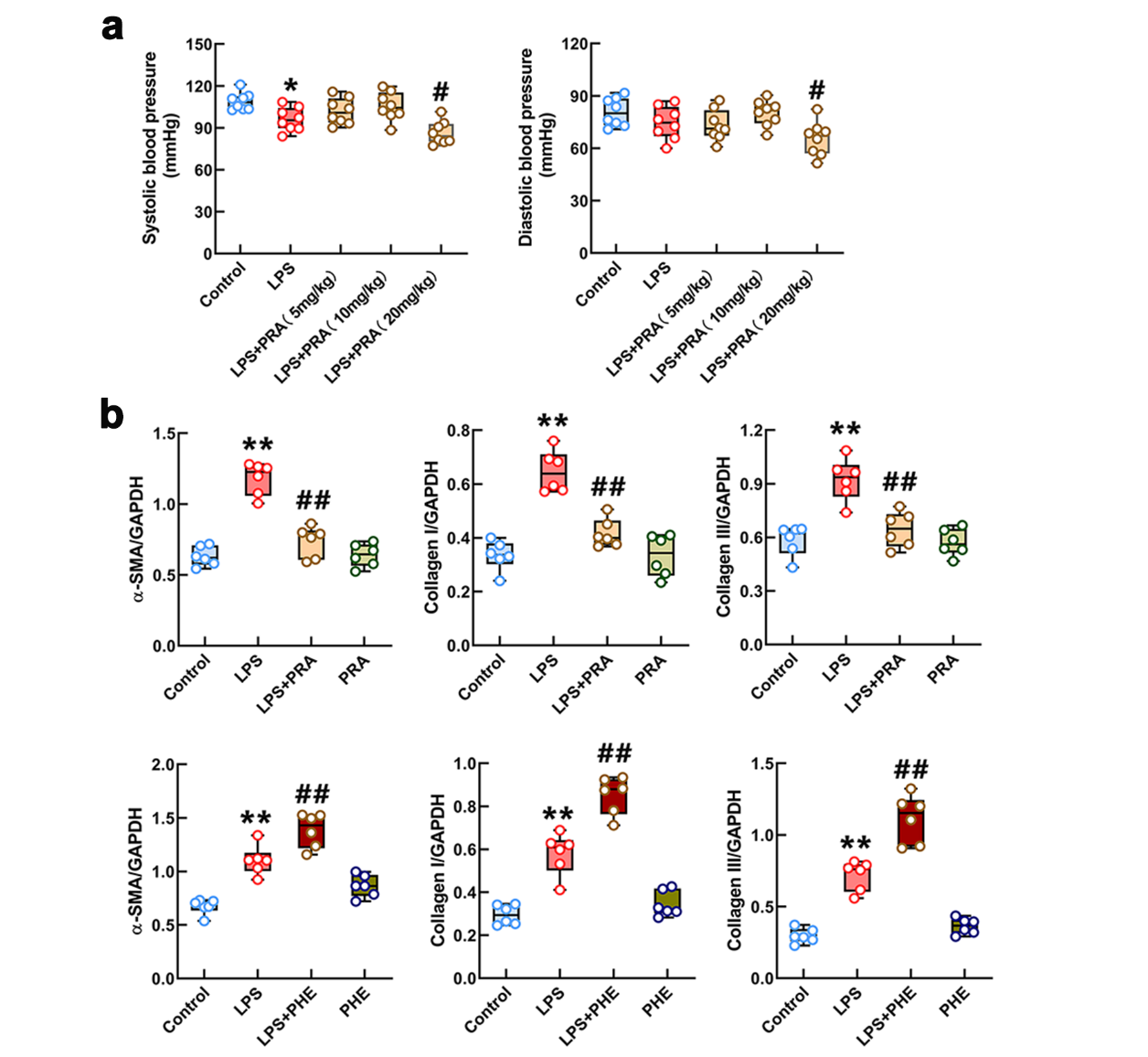
Fig. S1 Measurement of tail artery blood pressure and fibrotic biomarker proteins of myocardium in the mice of different groups. a** The effect of different doses of PRA on systolic (left) and diastolic (right) blood pressure in LPS-stimulated mice. **b** The quantitative statistical results of Western Blot assay for myocardial α-SMA and collagen I/Ⅲ levels in the LPS-injected mice treated with prazosin (PRA) or phenylephrine (PHE). Data are presented as mean ± SEM, n=6 or 8 in each group. ^*^*P* <0.05, ^**^*P* <0.01 vs Control group; ^#^*P* <0.05, ^##^*P* <0.01 vs LPS group.

**
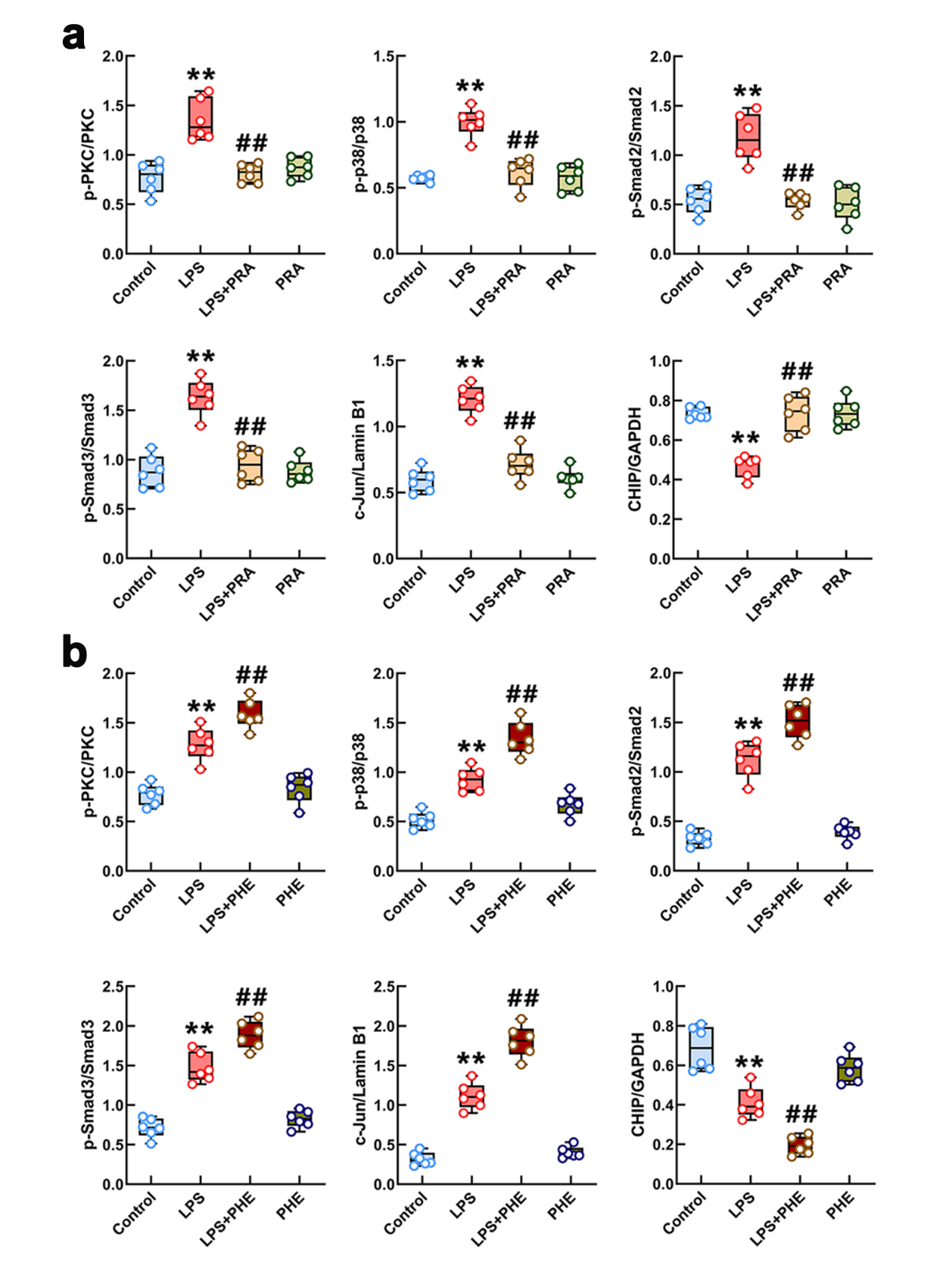
Fig. S2 Quantitative statistical results of Western Blot assay for the phosphorylation of PKC, p38 and Smad2/3, and the levels of CHIP and nuclear c-Jun in the myocardium of LPS-simulated mice treated with PRA (a) or PHE (b).** ^**^*P* <0.01 vs Control group; ^##^*P* <0.01 vs LPS group.

**
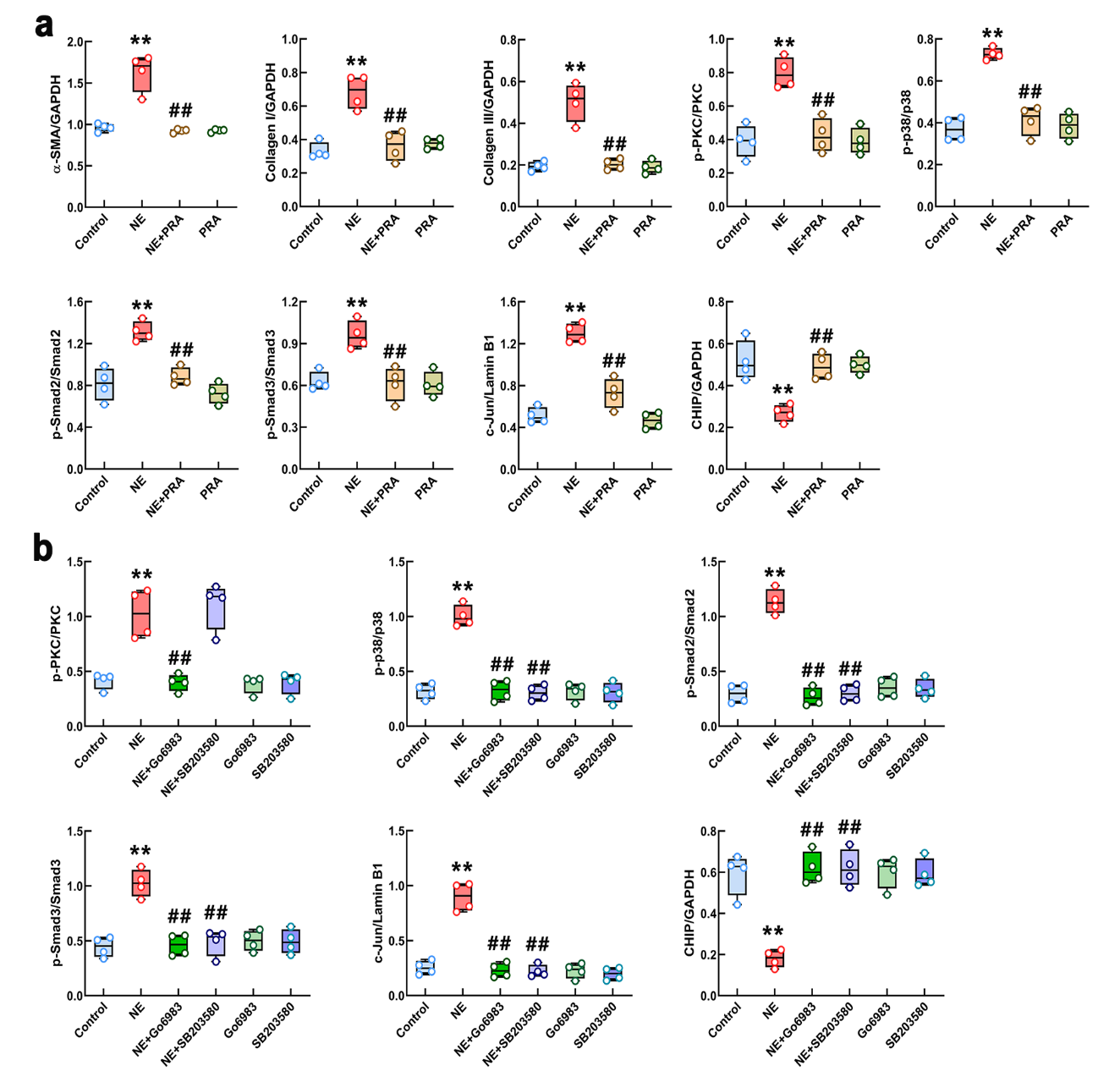
Fig. S3 Quantifications of the relative protein levels detected by Western Blot.** **a** The quantitative statistical results of Western Blot assay for α-SMA, Collagen I/Ⅲ, p-PKC, p-p38, p-Smad2/3, CHIP and nuclear c-Jun levels of the CFs in each group. **b** Quantifications of Western Blot assay for the PKC/p38/Smad2/3 phosphorylation, CHIP expression and nuclear c-Jun level in the NE-challenged CFs with Go6983 or SB203580 treatment. Data are presented as mean ± SEM, n=4 in each group. ^**^*P* <0.01 vs Control group; ^##^*P* <0.01 vs NE group.


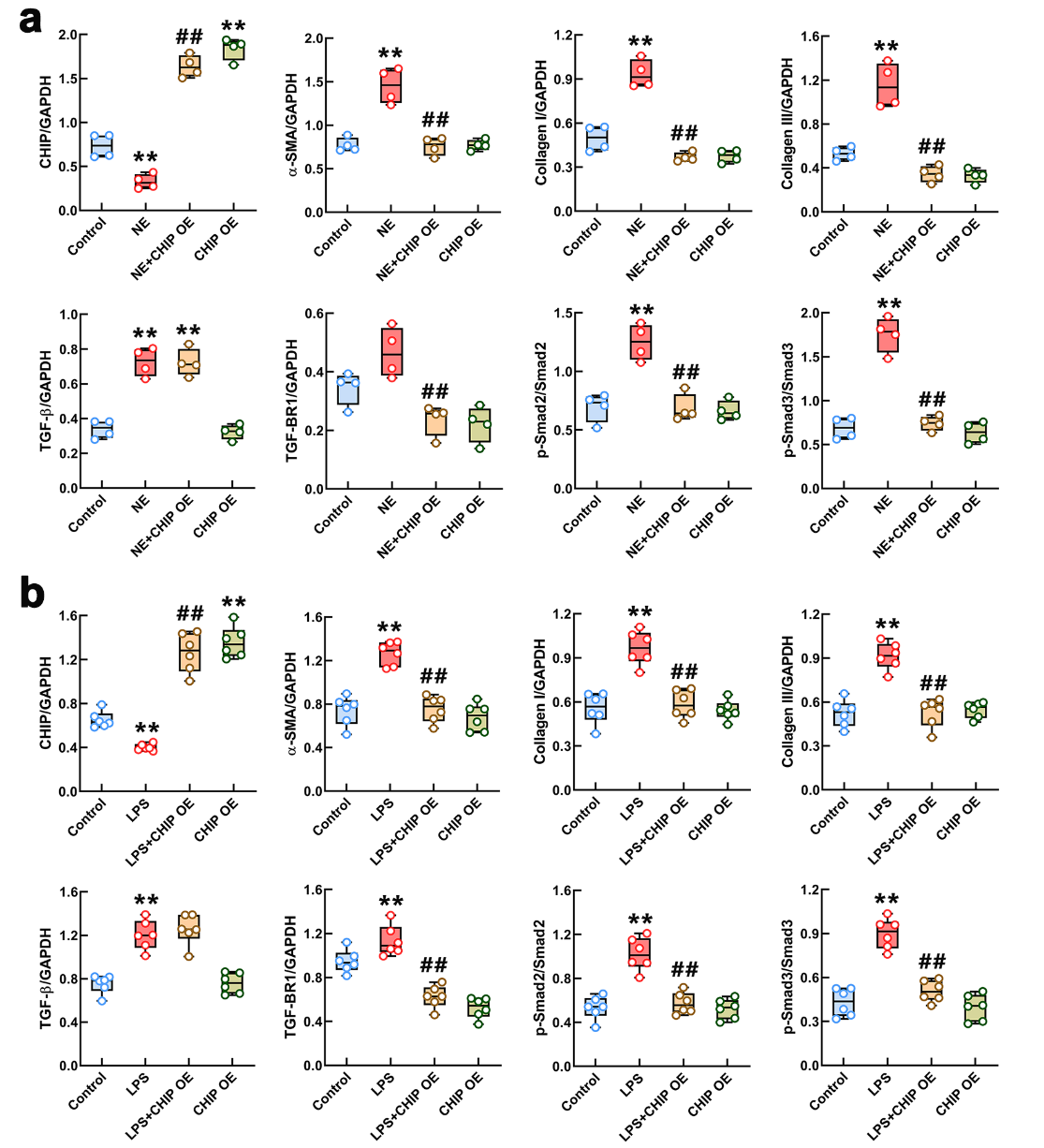
**Fig. S4 Quantifications of the relative protein levels detected by Western Blot. a** The quantitative statistical results of Western Blot assay for CHIP, α-SMA, collagen I/Ⅲ, TGF-β, TGF-BR1 and phosphorylation of Smad2/3 in the NE-stimulated CFs with or without CHIP overexpression (CHIP OE). **b** Quantifications of Western Blot assay for the proteins of myocardium in the LPS-stimulated mice with or without heart-specific CHIP overexpression. Data are presented as mean ± SEM, n=4 or 6 in each group. ^**^*P* <0.01 vs Control group; ^##^*P* <0.01 vs NE group or LPS group.

**
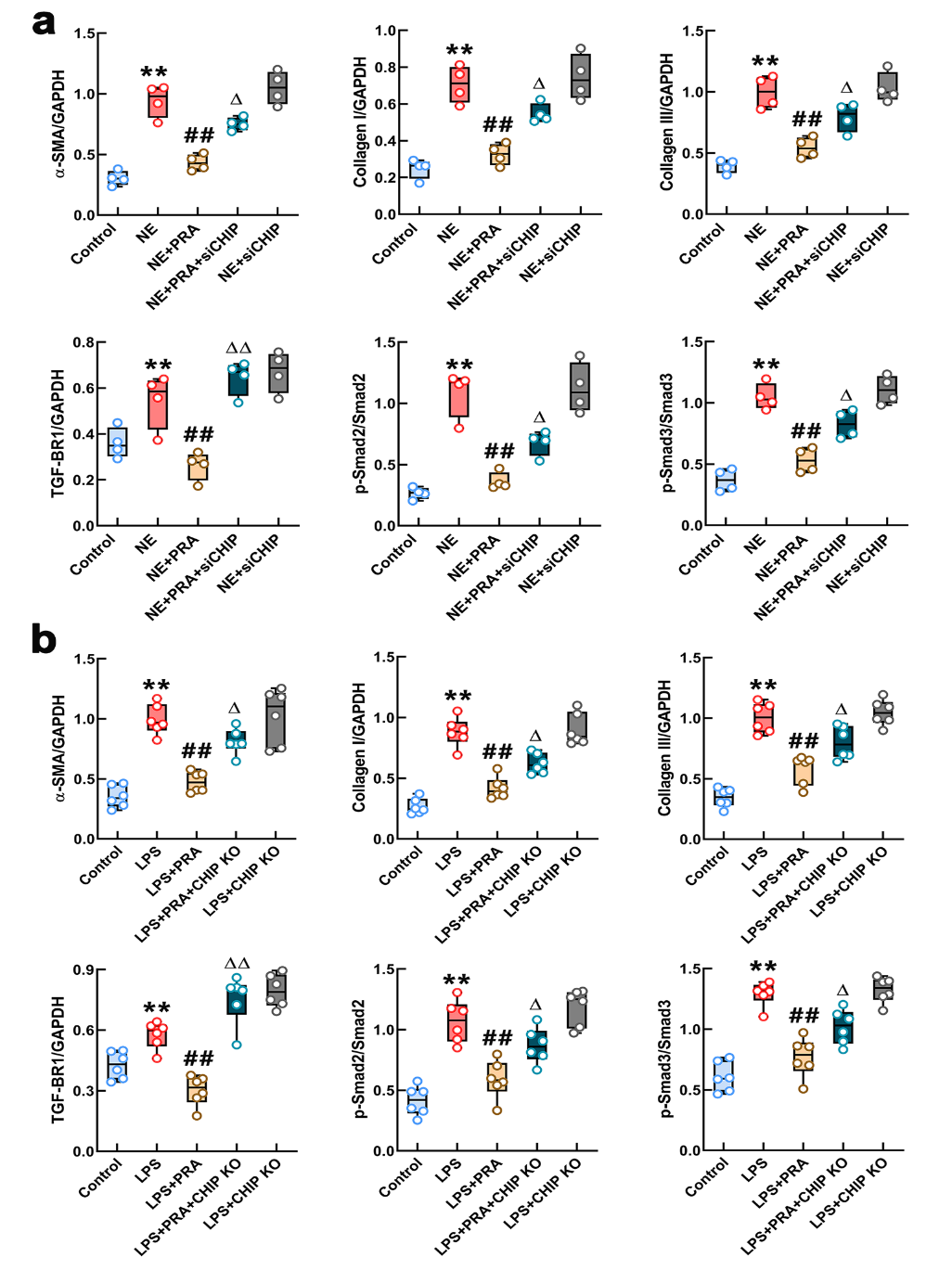
Fig. S5 Quantifications of the relative protein levels detected by Western Blot.** **a** The quantitative statistical results of Western Blot assay for the levels of α-SMA, collagen I/Ⅲ, TGF-BR1 and phosphorylation of Smad2/3 in the NE-stimulated CFs with or without CHIP knockdown (siCHIP). **b** Quantifications of Western Blot assay for myocardial levels of α-SMA, collagen I/Ⅲ, TGF-BR1 and phosphorylation of Smad2/3 in the LPS-treated mice with or without CHIP knockout (CHIP KO). Data are presented as mean ± SEM, n=4 or 6 in each group. ^**^*P* <0.01 vs Control group; ^##^*P* <0.01 vs NE group or LPS group. ^Δ^*P* < 0.05, ^ΔΔ^*P* < 0.01 vs NE+PRA+siCHIP group or L PS+PRA+CHIP KO group.

**
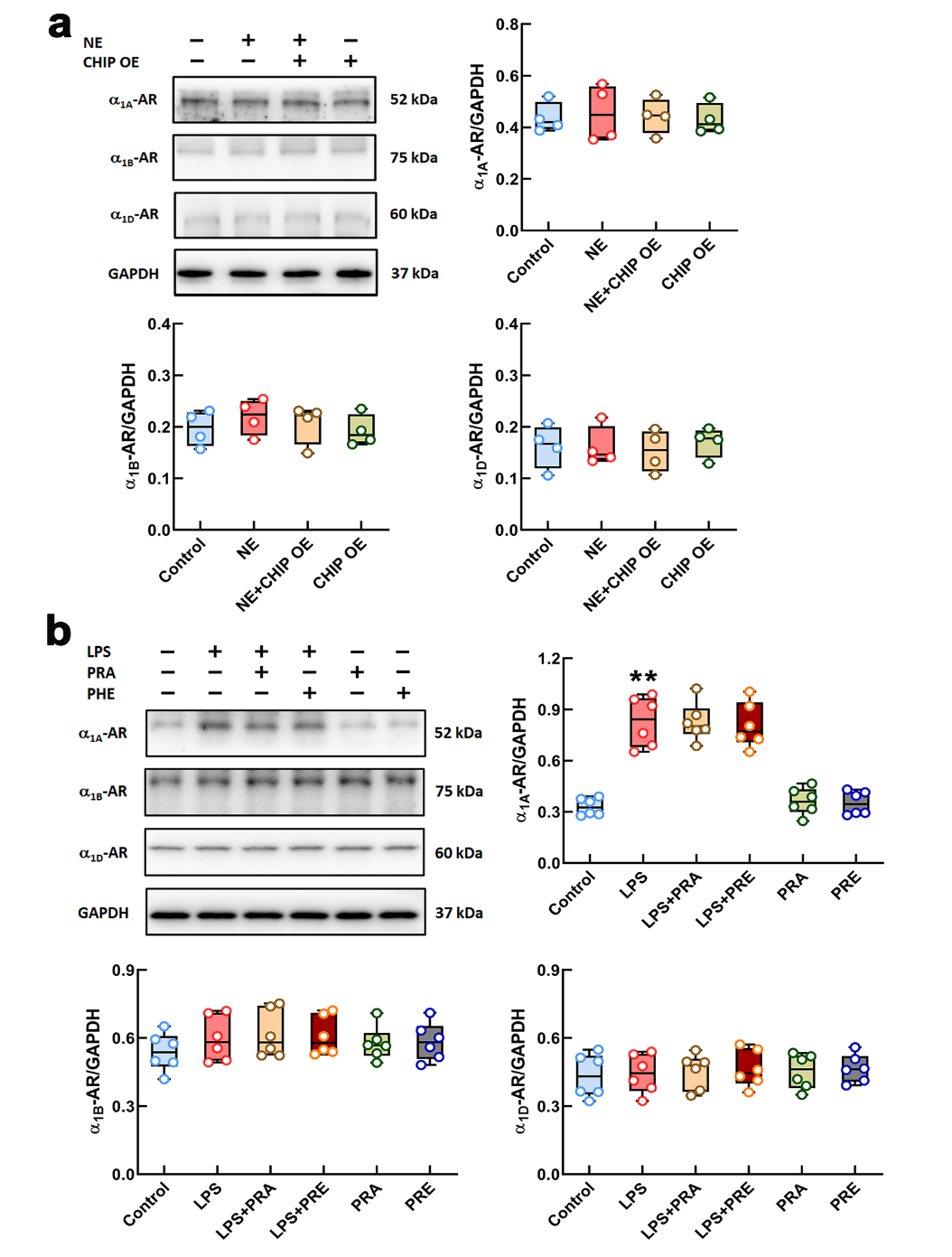
Fig. S6 CHIP overexpression, PRA and PHE have no effect on the expression of three α_1_**-**AR subtypes in the CFs or myocardium.** **a** The protein levels of α_1A_-AR, α_1B_-AR and α_1D_-AR in CFs of each group were measured by Western Blot. **b** The expressions of α_1A_-AR, α_1B_-AR and α_1D_-AR in the myocardium of mice in each group were assayed by Western Blot. Data are presented as mean ± SEM, n=4 or 6 in each group. ^**^*P* <0.01 vs Control group.


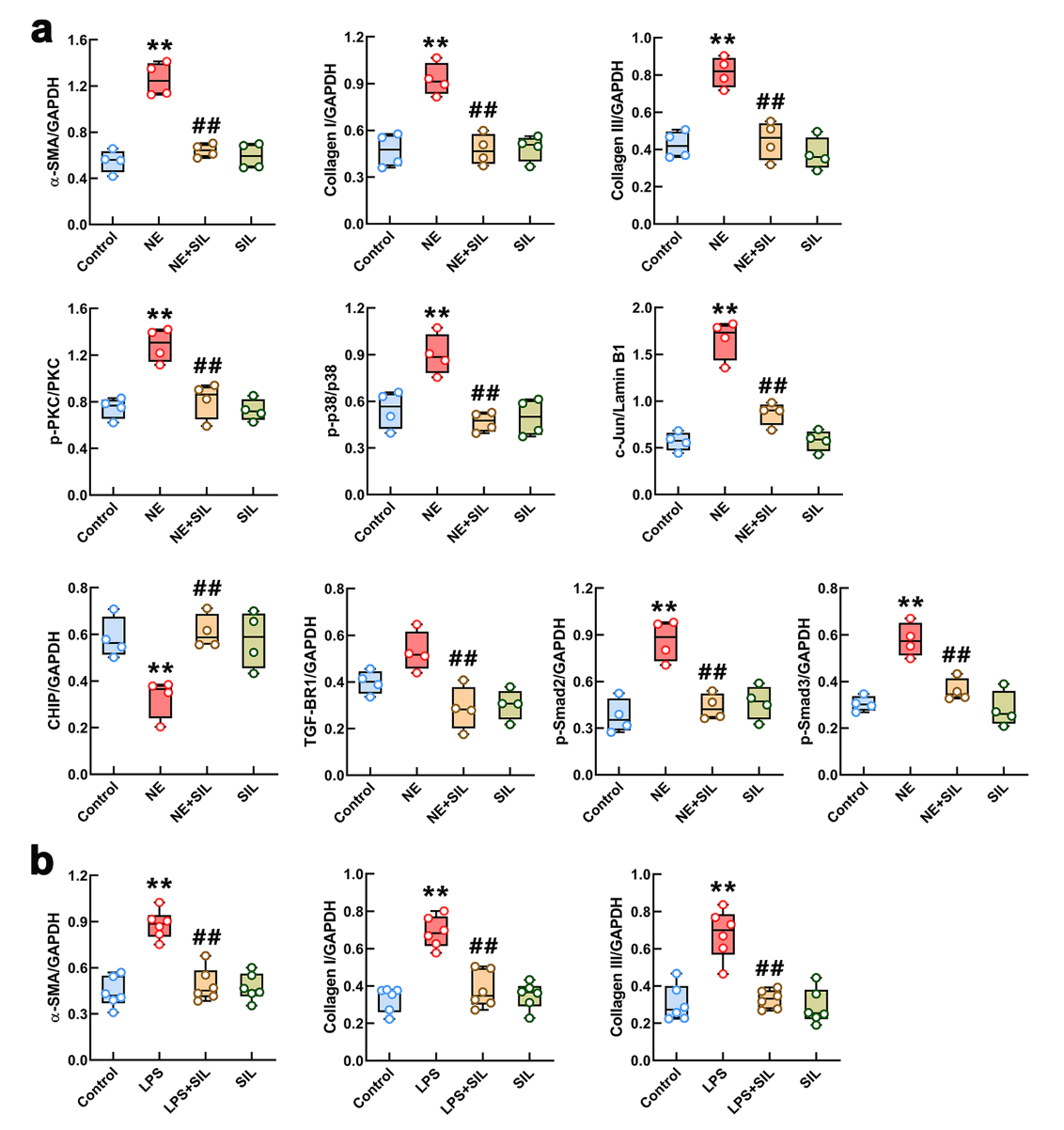
**Fig. S7 Quantifications of the relative protein levels detected by Western Blot.** **a** The quantitative statistical results of Western Blot assay for the levels of α-SMA, collagen I/Ⅲ, CHIP, TGF-BR1, nuclear c-Jun, and the phosphorylation of PKC, p38 and Smad2/3 in the NE-challenged cardiac fibroblasts with or without SIL treatment. **b** Quantifications of Western Blot assay for myocardial levels of α-SMA and collagen I/III in the mice of different groups. Data are presented as mean ± SEM, n=4 or 6 in each group. ^**^*P* <0.01 vs Control group; ^##^*P* <0.01 vs NE group or LPS group.
